# Exploring the Impact of Nanoparticle Stealth Coatings in Cancer Models: From PEGylation to Cell Membrane-Coating Nanotechnology

**DOI:** 10.1021/acsami.3c13948

**Published:** 2023-12-30

**Authors:** Pablo Graván, Jesús Peña-Martín, Julia López de Andrés, María Pedrosa, Martín Villegas-Montoya, Francisco Galisteo-González, Juan A. Marchal, Paola Sánchez-Moreno

**Affiliations:** † Department of Applied Physics, Faculty of Science, University of Granada, 18071 Granada, Spain; ‡ Department of Human Anatomy and Embryology, Faculty of Medicine, University of Granada, 18016 Granada, Spain; § Instituto de Investigación Biosanitaria de Granada (ibs.GRANADA), 18012 Granada, Spain; ∥ Biopathology and Regenerative Medicine Institute (IBIMER), Centre for Biomedical Research (CIBM), University of Granada, 18016 Granada, Spain; ⊥ Excellence Research Unit Modelling Nature (MNat), University of Granada, 18016 Granada, Spain; # BioFab i3D—Biofabrication and 3D (bio)printing laboratory, University of Granada, 18100 Granada, Spain; ¶ Faculty of Biology, Calzada de las Américas and University, Ciudad Universitaria, 80040 Culiacán, Sinaloa, Mexico

**Keywords:** nanoparticles, coatings, cell membranes, biointerfacing, protein corona, 3D cell culture

## Abstract

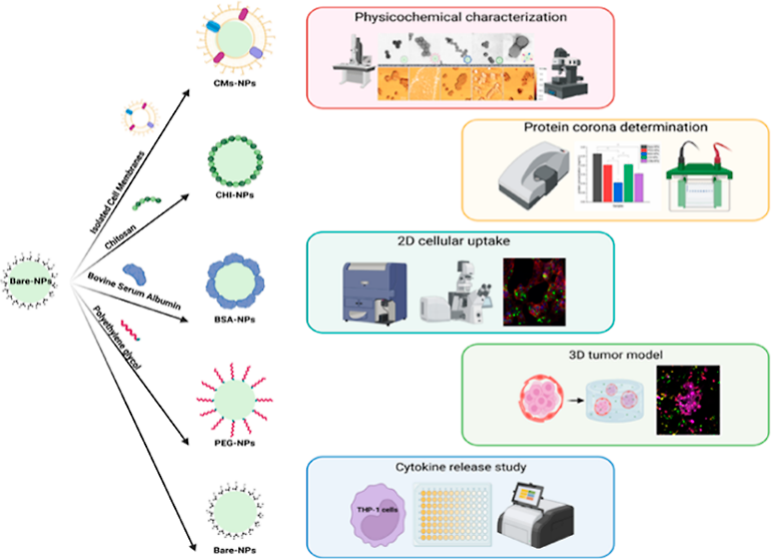

Nanotechnological platforms offer advantages over conventional therapeutic and diagnostic modalities. However, the efficient biointerfacing of nanomaterials for biomedical applications remains challenging. In recent years, nanoparticles (NPs) with different coatings have been developed to reduce nonspecific interactions, prolong circulation time, and improve therapeutic outcomes. This study aims to compare various NP coatings to enhance surface engineering for more effective nanomedicines. We prepared and characterized polystyrene NPs with different coatings of poly(ethylene glycol), bovine serum albumin, chitosan, and cell membranes from a human breast cancer cell line. The coating was found to affect the colloidal stability, adhesion, and elastic modulus of NPs. Protein corona formation and cellular uptake of NPs were also investigated, and a 3D tumor model was employed to provide a more realistic representation of the tumor microenvironment. The prepared NPs were found to reduce protein adsorption, and cell-membrane-coated NPs showed significantly higher cellular uptake. The secretion of proinflammatory cytokines in human monocytes after incubation with the prepared NPs was evaluated. Overall, the study demonstrates the importance of coatings in affecting the behavior and interaction of nanosystems with biological entities. The findings provide insight into bionano interactions and are important for the effective implementation of stealth surface engineering designs.

## 1 Introduction

Over the past few decades, nanoparticles (NPs) have become increasingly important in the field of nanomedicine owing to their unique physicochemical properties and potential applications, particularly in the treatment of tumors. Various types of NPs have been used for biomedical purposes, including liposomes and lipid-core NPs, polymeric NPs, and metallic NPs.^1^ NPs are able to overcome biological and physicochemical barriers in the body, allowing for the selective release of drugs and leading to an improved pharmacokinetic profile and reduced side effects associated with conventional therapeutic modalities.^2^ Despite the remarkable properties of NPs, efficient biointerfacing with the organism remains a major challenge for their in vivo application.^3^ While numerous NPs have been developed for cancer therapy, less than 1% of injected NPs are able to reach the tumor region, and only a few nanocarriers have been approved for clinical use.^4^

Once NPs enter the bloodstream, they face a complex environment designed to recognize and eliminate external entities. The first challenge is the interaction and adsorption of plasma proteins, such as serum albumin, immunoglobulins, and complement components, onto their surfaces, forming the protein corona. The formation of this structure alters the surface properties of NPs and plays a crucial role in their fate in a physiological environment. Moreover, components of the protein corona can activate the mononuclear phagocyte system and complement system, leading to rapid clearance of NPs from the bloodstream. Additionally, the biological corona can mask targeting ligands on the surface of NPs, resulting in reduced specificity in active-targeting strategies.^5^ Regardless of the therapeutic goals of NPs, prolonged circulation in the bloodstream is a fundamental requirement for effective in vivo drug delivery and therapeutic efficacy.^4^

To produce stealth NPs with longer circulation times, the traditional method is to coat them with nonionic surfactants such as poly(ethylene glycol) (PEG). Ethylene glycol units form a hydration layer by tightly associating with water molecules, preventing protein adsorption and subsequent clearance, which ultimately prolongs the circulation lifetime of the particles^6^ However, PEG is not able to completely prevent protein adsorption onto NPs, and therefore, the density and length of PEG molecules need to be adjusted to achieve optimal antifouling performance. Similarly, zwitterionic coatings are made up of molecules with balanced charges, which establish a neutral surface. These coatings strategically utilize structured layers to construct a water-based barrier that effectively prevents protein interference.^7^ However, the foreign nature of the synthetic antifouling polymers should be considered. For instance, an acquired immune-response to PEG moiety that compromises PEG-NPS performance has been reported.^8^

To address these limitations, biomacromolecules such as polysaccharides and proteins have also been used as coating materials for colloids due to their biocompatibility, biodegradability, and nontoxic properties.^9^ Dyopsonins, which include albumin and certain apolipoproteins, can shield NPs from phagocytosis, leading to an increased circulation time in the bloodstream and improved accumulation efficiency of NPs in other organs and tissues.^10^ For instance, albumin has been widely used as a protein-based coating material.^11^ Its excellent cost-effectiveness, good biodegradability, biocompatibility, and long plasma half-life have demonstrated great potential. On the other hand, chitosan (CHI), a natural polysaccharide derived from chitin, is a widely studied polysaccharide-coating material.^12^ CHI-coating can confer several advantages to NPs, including improved physicochemical stability, controlled drug release, modulation of cell interactions, and promotion of mucoadhesiveness. Both albumin and CHI are considered nontoxic materials that offer biocompatibility and biodegradability. Consequently, they are biomacromolecules widely employed in NPs intended for biomedical applications.

However, bottom-up fabrication strategies still struggle to replicate the multifactorial properties of biological systems and achieve efficient biointerfacing. In the past decade, cell membrane (CM)-coating nanotechnology has emerged as a top-down biotechnology to produce stealth nanosystems. First reported in 2011 by Hu et al.,^13^ this method involves isolating and transferring the CM of a cell directly onto the surface of a particle. The resulting CM-coated NP inherits the proteins, carbohydrates, and lipids from the source cell, resulting in improved abilities to interface with physiological environments such as targeting capabilities and longer circulation times.^14^ The emerging technology of the CM coating has become a novel concept for the design of NPs, and it has been leveraged to significantly improve the functionality of nanoparticulate platforms. However, this line of research has occurred in the absence of any complete and comparative study using well-developed and trustworthy physicochemical characterization techniques that allow us to understand the benefits and drawbacks of this type of stealth coating. Moreover, in this research field, the most effective method for membrane extraction before coating of NPs remains unclear.

It is widely recognized that the surface of a nanosystem, which refers to the outermost layer of the material, plays a critical role in determining its physical, chemical, and biological properties. Furthermore, coatings have a significant impact on how the nanosystem interacts with its environment and will affect the delivery journey of NPs in vivo.^6^ Understanding the importance of surface and coating properties in the behavior of NPs is crucial for the development of safe and effective nanotechnologies.

In this work, we aim to perform a comparative study on the behavior of NPs coated with different stealth coatings broadly described in the literature (including PEG, proteins, and polysaccharides) and the novel concept of the bionic CM-coating technology literature. Specifically, we prepared differently coated NPs with a common polystyrene core, including PEG-NPs, bovine serum albumin (BSA)-NPs, CHI-NPs, and NPs coated with CMs extracted from the human breast adenocarcinoma cell line MCF-7. In addition, we have compared all of the methodologies that have been explored for the extraction of CMs and have selected the best one to carry out the coating of NPs.

We evaluated the colloidal behavior of the prepared NPs and their interactions with biological systems to assess the importance of the surface and how it affects their performance. NPs were physicochemically characterized, and their interactions with serum proteins and subsequent formation of a protein corona were evaluated. We studied the comparative incorporation of the prepared particles inside MCF-7 tumor cells using flow cytometry and confocal optical microscopy techniques. In addition, we employed heterogeneous multicellular spheroids of MCF-7 tumor cells and fibroblasts (FBs) embedded in a type-I collagen matrix to study the behavior of the prepared NPs in a biomimetic tumor context. 3D tumor models have proven to be a representative platform for the study of pharmacological responses and NP uptake since they more closely recapitulate the characteristics of native tumors.^15^ Specifically, extracellular matrix (ECM)-derived hydrogels provide an aqueous environment in which different cell types can proliferate and interact with their surrounding cells and matrix.^16^ Multicellular spheroids allow complexing the models by including stromal cells, replicating cell–cell interactions, and providing more accurate tumor conditions.^17^ Additionally, since tumors have the ability to suppress the immune system, an inflammatory response is crucial for tumor treatment. In this sense, we also tested the ability of the prepared NPs to trigger an inflammatory response on the human monocyte THP-1 cell line by studying the secretion of pro-inflammatory cytokines.

## 2 Materials and Methods

### 2.1 Reagents

100 nm yellow-green FluoSpheres carboxylate containing fluorescein isothiocyanate (FITC) was purchased from ThermoFisher (Madrid, Spain). BSA, 1-ethyl-3-(3-(dimethylamino)propyl) carbodiimide (EDC), sulfo-*N*-hydroxysulfosuccinimide (Sulfo-NHS), *O*-(2-aminoethyl)polyethylene glycol 3000 (NH_2_-PEG) and CHI, low-molecular-weight (CAS: 9012-76-4), and lipopolysaccharide (LPS) were purchased from Sigma-Aldrich (Madrid, Spain). All aqueous solutions were prepared using ultrapure water from a Millipore Milli-Q Academic pure-water system.

### 2.2 Cell Lines and Culture Conditions

MCF-7 human breast cancer and human monocyte THP-1 cell lines were obtained from the American Type Culture Collection (ATCC). Human dermal FB (HDFa) cell line was purchased from ThermoFisher scientific. MCF-7 cells were cultured in Dulbecco’s modified Eagle’s medium (DMEM) supplemented with 10% (v/v) heat-inactivated fetal bovine serum (FBS) (Gibco), 1% l-glutamine, 2.7% sodium bicarbonate, 1% HEPES buffer, and 1% penicillin/streptomycin solution (GPS, Sigma). FBs were cultured in Human Fibroblast Expansion medium (ThermoFisher scientific). THP-1 cells were cultured in the Roswell Park Memorial Institute (RPMI) culture media. Cells were grown at 37 °C in an atmosphere containing 5% CO_2_ and 95% humidity. Cell lines were tested routinely for mycoplasma contamination.

### 2.3 CM Isolation

For CM derivation, MCF-7 cells were grown in T-175 culture flasks to full confluency and physically detached with a scraper in phosphate-buffered saline (PBS). Cells were collected and washed in PBS three times by centrifugation at 500*g* for 5 min. Then, cells were suspended in a hypotonic lysis buffer consisting of 10 mM tris-HCl pH 7.4, 1 mM KCl, 25 mM sucrose, 1 mM MgCl_2_, 10 μg/mL of DNase and RNase, and EDTA-free protease inhibitor. Cells were disrupted using a Dounce homogenizer with a tight-fitting pestle under ice-cold condition. The solution was centrifuged at 600*g* for 5 min. The postnuclear supernatant (PNS) was saved, while the pellet was resuspended in hypotonic lysis buffer and subjected to further homogenization and centrifugation. Three centrifugation protocols were adapted and compared to isolate CMs from the collected PNS. For the first protocol, namely, PLT17, the PNS was centrifuged at 17,000*g* for 30 min. The generated pellet was considered as the CM fraction.^18,19^ The second method consisted in a first centrifugation of the PNS at 17,000*g* for 30 min in which the pellet is discarded and the supernatant (SN17) is further ultracentrifuged at 100,000*g* for 1 h.^13,20,21^ This final pellet is collected as CMs. For the third protocol, a discontinuous sucrose gradient centrifugation method was followed.^22,23^ Briefly, the collected PNS was placed on a sucrose gradient (55–40–30% w/v sucrose) in polycarbonate tubes and centrifuged at 28,000*g* for 45 min at 4 °C in a Beckman SW 28 rotor. The lipid-rich fraction at the 30–40% interface was collected, washed, and centrifuged at 28,000*g*. The final membrane-rich pellets obtained after each protocol were collected and stored for subsequent experiments. Membrane content was quantified indirectly by measuring the protein content of the samples using a BCA kit (Pierce) in reference to a BSA standard.

### 2.4 Western Blotting

CMs were further characterized by Western blotting. For sodium dodecyl sulfate–polyacrylamide gel electrophoresis (SDS-PAGE), 10 μg of protein from each sample was mixed with loading buffer [62.5 mM tris-HCl (pH 6.8 at 25 °C), 2% (w/v) SDS, 10% glycerol, 0.01% (w/v) bromophenol blue, and 40 mM dithiothreitol]. Then, the samples were boiled for 5 min, and an equal sample volume was loaded into each well of a 4–20% polyacrylamide gel (Mini-PROTEAN TGX) using an electric field of 150 V and a Mini-PROTEAN Tetra electrophoresis system from BioRad. Subsequently, the proteins were transferred to nitrocellulose membranes (Whatman) using an XCell II Blot Module (Invitrogen) and transfer buffer (Invitrogen), following the manufacturer’s instructions. The membranes were probed with an antibody cocktail (ab140365, abcam) against sodium potassium ATPase, GRP78, ATP5A, GAPDH, and Histone H3, along with a horseradish peroxidase-conjugated antirabbit IgG (sc-2357, Santa Cruz). The films were developed using an ECL Western blotting substrate (Pierce) and a Mini-Medical/90 Developer (ImageWorks).

### 2.5 Preparation of Coated NPs

Carboxylate-modified polystyrene FluoSpheres were used as the core of all the coated NPs. BSA and PEG were immobilized onto the surface of the NPs through a carbodiimide reaction. EDC and S-NHS were employed to achieve covalent binding through the carboxylic acid groups present on the NPs and the amine groups of the BSA and NH_2_-PEG.^24^ Briefly, 50 μL of NPs (1 mg) was made to react in MES buffer (pH 5.5) with 1 mg of EDC and 2.4 mg of Sulfo-NHS for 20 min at RT. Subsequently, the NPs were centrifuged at 20,000*g* for 30 min. Furthermore, the NPs were resuspended in borate buffer (pH 8) and incubated with 5.6 mg of NH_2_-PEG or 3.4 mg of BSA for 1 h. CHI was coated onto the surface of the NPs by electrostatic deposition under acidic conditions, where ionic attraction occurs between the cationic ammonium groups in the chitosan and the carboxylate groups on the NPs.^25^ Briefly, 50 μL (1 mg) of NPs was incubated in a 1% acetic acid aqueous solution (pH 4) at a final CHI concentration of 0.01%. The reaction was allowed to proceed with proper agitation for 1 h at RT. Membrane-coated NPs were prepared at a membrane to polymer ratio of 1:1, according to a previously described protocol.^26^ CM coating was carried out by mixing 1 mg of NPs with 1 mg (protein) of membranes and sonicating the mixture for 3 min in a bath sonicator operating at 50/60 Hz and 360 W (JP Selecta 3000513). After each coating procedure, samples were cleaned by centrifugation to remove noncoupled and excess molecules and resuspended in a phosphate buffer solution (pH 7). The concentration of NPs was quantified based on the FITC fluorescence in their core (*R* > 0.99) (Figure S10).

### 2.6 Characterization of NPs

For the physicochemical characterization, the hydrodynamic diameter (*D*_h_), polydispersity index (PDI), and z-potential were determined by dynamic light scattering (DLS). The self-optimization routine in Zetasizer software was used for all measurements, and the z-potential was calculated according to the Smoluchowsky theory. Samples were diluted with a low-ionic-strength phosphate buffer (1.13 mM KH_2_PO_4_, pH 7) and measured at 25 °C in triplicate. Results appear as the mean value ± standard deviation (SD). To assess the pH impact, NPs were diluted (1:100) in a low-ionic-strength (<2 mM) buffer solution with the desired pH value and then incubated for 30 min before measuring. Similarly, to study the ionic strength, solutions of increasing concentrations of KNO3 were employed at a fixed pH of 7. To determine the critical coagulation concentration (CCC) of the colloidal solution, the Fuchs factor (*W*) was calculated using a Beckman DU 7400 spectrophotometer, as previously described.^27^ KNO_3_ was employed as the salt solution.

The morphological analysis was performed by using transmission electron microscopy (TEM) and atomic force microscopy (AFM). To perform TEM imaging, 25 μL of each sample was incubated on carbon-coated grids for 5 min and then washed with ultrapure water. Negative staining was conducted using uranyl acetate. Subsequently, grids were dried on filter paper and observed in a high-resolution TEM (HRTEM) TITAN from FEI Company operated at 300 kV. For AFM analysis, an NX-20 instrument (Park Systems, Suwon, Korea) was used. Each sample was diluted in H_2_O Milli-Q at a concentration of 0.1 mg/mL and deposited onto freshly cleaved muscovite mica for 10–15 min. Then, the samples were rinsed three times with Milli-Q water (Millipore, Burlington, MA, USA) to remove salts and loosely bound NPs and dried before imaging with a gentle stream of argon. Details of AFM nanomechanical properties analysis can be found in the Supporting Information.

SDS-PAGE was employed for the protein characterization of BSA-NPs and CMs-NPs. The gels were silver stained using a 2D Silver Stain Kit II (Cosmo Bio Co., Ltd.) and analyzed with image J (1.410 version). To assess the functionalization of PEG onto the surface of PEG-NPs, NMR spectra were recorded on a Bruker AVANCE III spectrometer (500 MHz for ^1^H) equipped with a 1.7 mm MicroCryoprobe using external acetone referencing for the analysis of intact PEG-NPs in H_2_O–D_2_O. The grafting density of PEG molecules onto the NPs was determined using a colorimetric assay described by Baleux.^28^ Briefly, 25 μL of an iodine–potassium iodide solution (0.4 M I2, 0.12 M KI) was added to 1 mL of the different samples. After 5 min of incubation, the optical density (OD) of the solutions was measured at a wavelength of 500 nm. The samples were previously diluted to ensure accurate measurements within an optimal adsorption range (0.1 < AU < 1.0). A calibration curve was established using free PEG, enabling the conversion of the measured OD values into quantitative PEG concentration data for the samples (Figure S1E). Similarly, the BCA method was employed to calculate the grafting of BSA onto the surface of NPs.

### 2.7 Protein Corona Determination

To study the formation of the protein corona, the NPs were dispersed in 1 mL of complete DMEM with 10% FBS and incubated at 37 °C for 1 h. To obtain the corona–NP complexes, the NPs were centrifuged at 20,000*g* for 30 min at 4 °C to remove unbound proteins. The obtained pellet was then washed with PBS under the same conditions. To study the change in the colloidal properties of the corona–NP complexes, the hydrodynamic diameter (*D*_h_), PDI, and z-potential were measured in low-ionic-strength (<2 mM) buffer solutions of pH 4, 7, and 9. The BCA assay was used to determine the amount of protein adhered to the NPs after incubation. Triplicates were used.

### 2.8 Cellular Uptake of Coated NPs

Cellular uptake of coated NPs by MCF-7 cells was assessed by flow cytometry and confocal fluorescence microscopy. For the flow cytometry assay, 1 × 10^5^ cells were seeded into 12-well culture dishes and treated with 10 μg/mL of each NPs for the selected time points (1.8 × 10^5^ NPs/cell). Then, cells were detached, centrifuged at 500*g* for 5 min, washed with PBS twice, resuspended in 300 μL of PBS, and analyzed by flow cytometry with an FACS Canto II instrument (FACSCanto II, Becton Dickinson, New Jersey, US) using the software FACSDiva 6.1.2 (Becton Dickinson) for data analysis. Confocal microscopy images were taken with a Leica DMI6000 inverted laser confocal microscope. 1 × 10^5^ MCF-7 cells were seeded in 13 mm tissue culture coverslips purchased from Sarstedt (Newton, NC). After 24 h, the culture medium was changed, and cells were incubated with 10 μg/mL of NPs for 36 h. Cells were washed twice with prewarmed PBS and fixed for 10 min with a 4% PFA solution in PBS for 10 min. Coverslips were then treated with a 0.1% Triton X-100 solution for 5 min and, subsequently, incubated during 30 min in 1% BSA–PBS. Fixed cells were incubated for 30 min with Alexa Fluor 647 phalloidin for F-actin staining and finally for 5 min with Hoestch for nucleus visualization. Samples were washed twice with prewarmed PBS after each step. All experiments were performed in triplicate.

### 2.9 Cellular Uptake on Multicellular Spheroids

Multicellular spheroids were formed by using the hanging drop technique. Briefly, MCF-7 cells were stained with CellTracker Deep Red (ThermoFisher) as per the manufacturer’s instructions and seeded at a concentration of 500 cells/drop in culture medium containing methylcellulose (2.4 mg/mL, Sigma-Aldrich) on top of a Petri dish lid, which was then cultured inverted. After 24 h, FBs were stained with CellTracker Red (ThermoFisher) and seeded under the same conditions at a concentration of 500 cells/drop over the MCF-7 spheroids. After another 24 h, MCF-7-FBs spheroids were collected and centrifuged. A solution of type-I collagen (3.58 mg/mL rat tail collagen, Corning) was neutralized with 1 M NaOH, mixed with the cell pellet, seeded into 48-well plates, and gelled at 37 °C for 30 min. Then, fresh media were added and replaced after 72 h. The final volume of each hydrogel was 200 μL, and the cell density was 1 × 10^6^ cells/mL. Hydrogels were treated with 10 μg/mL NPs. After 36 h, NP uptake was analyzed by confocal microscopy (Leica TCS SP2). Colocalization of NPs and cells was analyzed by using the software ImageJ.

### 2.10 Multiplex Cytokine Analysis

Cell culture supernatants from THP-1 cells treated with 10 μg/mL NPs for 24 h were used to quantify the secretion of cytokines using a Multiplex Human Cytokine enzyme-linked immune sorbent assay (ELISA) Kit (MBS590064, MYBioSource) according to the manufacturer’s protocol. The cell supernatants were collected and briefly centrifuged to remove the cellular debris prior to cytokine analysis. LPS at a concentration of 1 μg/mL was used as a positive control. The following human cytokines were measured: IL-1, IL-6, GM-CSF, MACF, and TNF-α. Final concentrations were calculated from the mean fluorescence intensity and expressed in picograms per milliliter. All incubation steps were performed at room temperature and in the dark.

### 2.11 Statistical Analysis and Representation

The obtained data were analyzed using Origin software (OriginLab Corporation, Northampton, Massachusetts, USA). Data appears as the mean value ± standard deviation. Data pairs were analyzed with one-way analysis of variance (ANOVA) with Tukey mean comparison method (*p* < 0.05).

## 3 Results and Discussion

### 3.1 Comparison of CM Isolation Protocols

**Figure 1:**
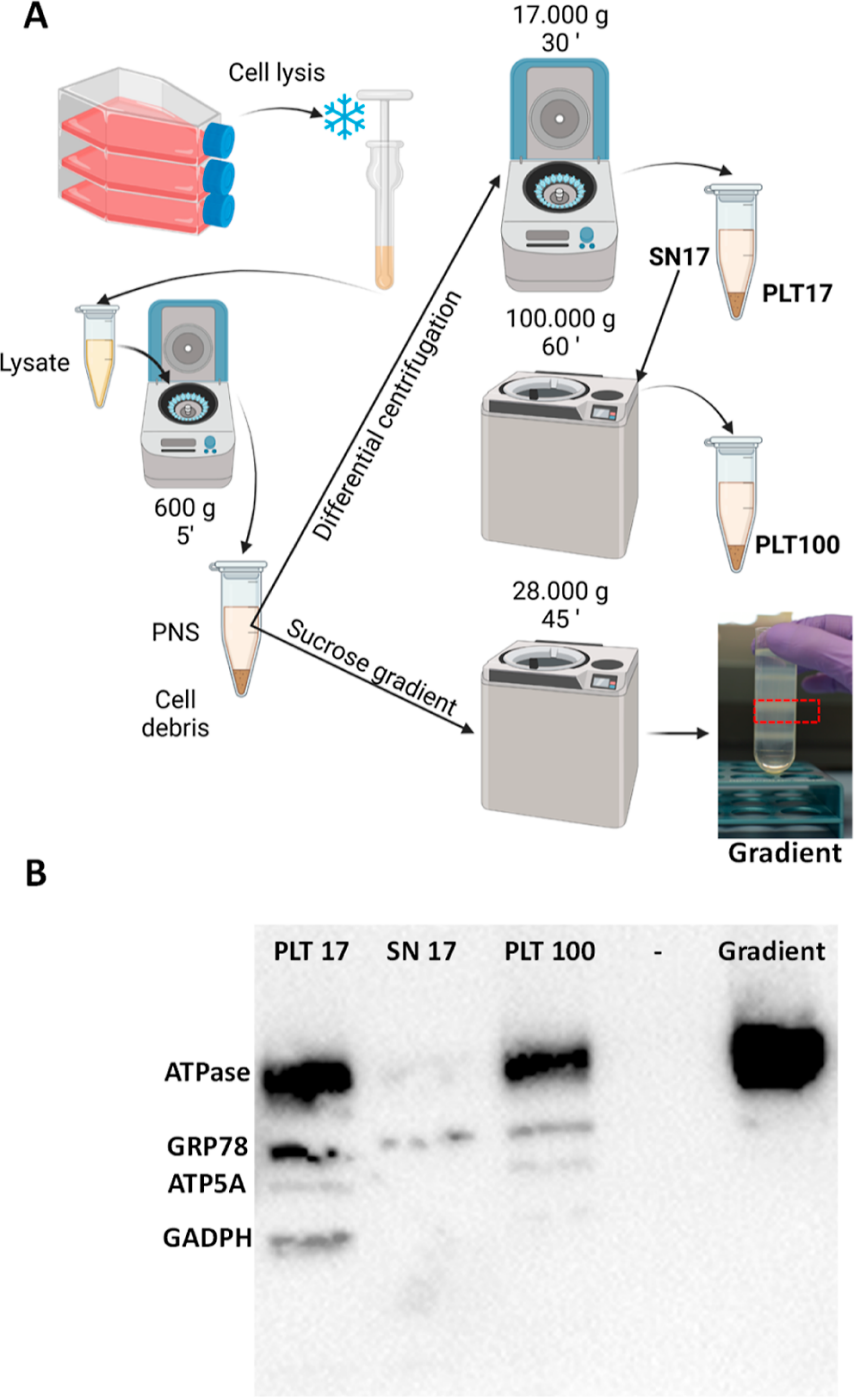
(A) Schematic representation of the three different protocols to isolate CMs: PLT17, PLT100, and gradient, created with BioRender.com and (B) Western-blot analysis of the CMs obtained from the three different methods.

In recent years, CM nanotechnology has emerged as an alternative to conventional strategies for producing stealth nanosystems. In this top-down technology, isolated CMs are employed to coat the surfaces of NPs. However, a lack of consensus exists regarding the method employed to extract CMs. The CM isolation process begins with a common first step, where cells are lysed, usually with a hypotonic lysis buffer and a Dounce homogenizer. This lysate is then centrifuged between 400 and 1000*g* to obtain the PNS, which is free of nucleus and cell debris. After this step, three different centrifugation protocols are found in the literature to isolate CMs from the PNS. The simplest protocol consists of differential centrifugation of the PNS between 14,000 and 20,000*g* for 20–30 min, after which the pellet is saved as the isolated CMs.^18,19^ However, we also found a protocol in the literature that discards this first pellet and further ultracentrifuges the obtained supernatant at 100,000 for 30–60 min.^13,20,21^ In the last methodology, the obtained PNS is placed on a discontinuous sucrose gradient (55–40–30% w/v sucrose) and centrifuged at 28,000*g* for 30–45 min. The band deposited at the 30–40 interface is collected, washed, and further centrifuged to obtain isolated CMs.^22,23^ Within this scenario, given the inconsistency presented between the two methods of differential centrifugation, in which some authors use the first pellet as the CM fraction, whereas others discard this pellet and further ultracentrifuge the supernatant, we tested the three described protocols to obtain CMs from the MCF-7 cell line (Figure 1A). The obtained CMs from the different protocols, namely, pellet 17,000*g* (PLT17), pellet 100,000*g* (PLT100), and gradient, were analyzed by Western blotting for a series of membrane and intracellular protein markers: plasma membrane-specific marker (Na^+^/K^+^-ATPase), endoplasmic reticulum marker (GRP78), mitochondrial maker (ATP5a), and cytosol marker (GAPDH) (Figure 1B). As can be seen in the figure, the discontinuous sucrose gradient protocol achieved the greatest enrichment in the membrane marker, while markers from the endoplasmic reticulum, mitochondria, and cytosol were not present, indicating negligible contamination from the subcellular organelles. PLT17 and PLT100 showed a lower amount of the CM marker, while the other markers were present. Based on these results, we decided to employ the sucrose-gradient method to isolate CMs to produce CMs-NPs. Nonetheless, although the sucrose gradient protocol achieves the isolation of the purest CMs, the least amount of CMs is obtained. We have observed that, starting with the same MCF-7 cell number, the PLT100 and PLT17 protocols achieve 3× and 1.5× higher amounts of CMs (milligrams of protein) than the sucrose-gradient method, respectively. Furthermore, these methods are extensively employed and should also be considered to extract bioactive CMs.

### 3.2 Size, z-Potential, and Morphology of the Prepared NPs

**Figure 2:**
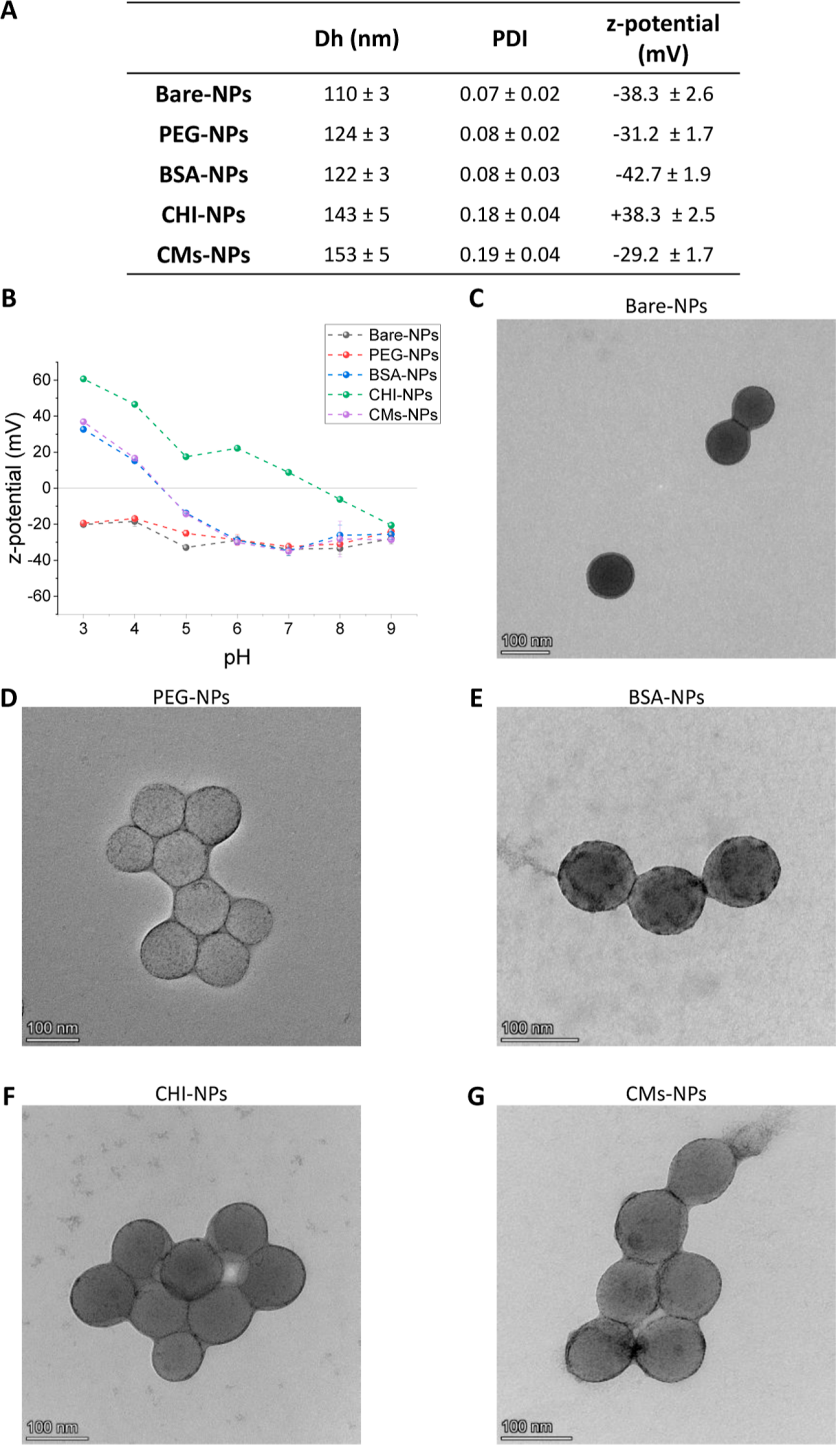
(A) Table presenting the *D*_H_, PDI, and z-potential of the prepared NPs, (B) z-potential depending on the pH of the media, and TEM micrographs obtained in bright-field mode of uncoated (C) and coated NPs (D–G), PEG-NPs, BSA-NPs, CHI-NPs, and CMs-NPs, respectively.

Different approaches were carried out to prepare the coated NPs. PEG and BSA were covalently linked onto the surface of carboxylated NPs via *carbodiimide* reaction.^24^ CHI was deposited onto the surface of NPs by electrostatic interaction.^25^ For CMs-NPs, CMs were collected following a discontinuous sucrose gradient ultracentrifugation protocol, and coating was performed by sonication. Bare and coated NPs were physicochemically characterized in terms of *D*_H_ and z-potential by DLS, and their morphology was studied by TEM. The effect produced by the different coatings of the nanosystems was reflected in the properties studied (Figure 2). An increase in the size of the coated NPs, especially evident in the CMs-NPs, was observed. The *D*_H,_ PDI and z-potential of the prepared NPs are presented in Figure 2A. The analysis of the surface potential along the range of pH demonstrates the different behaviors of the prepared nanosystems based on their coating. NPs coated with the polycation CHI possessed a positive surface potential in almost the entire pH range tested, showing their isoelectric point (IEP) around pH 8, as reported in the literature.^29^ BSA-NPs and CMs-NPs change from a positive surface potential at acidic pHs to a negative potential at higher pH. The IEP of BSA- and CM-NPs match with the already reported data^30,31^ This electrophoretic behavior is typical of proteins. At basic pH values, amino and carboxyl groups are protonated, which gives the system a positive net charge, whereas at acidic pH, carboxyl groups are negatively charged, turning the net surface potential of the system. On the other hand, bare NPs and PEG-NPs showed a relatively constant negative surface potential along the pH range tested.

TEM micrographs confirmed the size of the NPs and showed their spherical shape (Figures 2C–G and S2). Visual differences in the appearance of the NPs, mainly on their surface, were observed. This effect is possibly due to the different interactions of each type of coating with the negative staining employed during sample preparation. Furthermore, to confirm the presence of BSA and CMs on the surface of the NPs, SDS-PAGE was employed (Figure S1A). Western blotting was also employed to confirm the presence of membrane markers on the prepared CMs-NPs (Figure S1B). To confirm the cloaking of PEG, NMR was employed to study the ^1^H NMR spectrum and diffusion-ordered NMR spectrum of intact and purified PEG-NPs (Figure S1C,D), observing signals corresponding to PEG. In addition, PEG grafted on the surface of the NPs was measured through a colorimetry assay (*R*^2^ > 0.99, Figure S1E). For PEG, a calculated value of 8068 ± 401 PEG molecules per NP was obtained, corresponding to 73.1 ± 3.7 μg of PEG/mg of NPs. Compared to the literature, these values align with PEGylated NPs falling within the medium–low range of PEG grafting density.^32,45^ Similarly, the BCA method was employed to calculate BSA grafting (Figure S1E), yielding 2036 ± 6 BSA molecules per NP (413.27 ± 1.27 μg of BSA/mg of NPs).

### 3.3 Colloidal Stability

Colloidal stability is a limiting aspect in the industrial development of NPs. DLVO theory describes the stability of colloids, with electrostatic repulsion being the main phenomena that keeps the nanosystem kinetically stable.^33^ The surface electrical state of a colloidal system is dependent on the surface composition and composition of the medium. The colloidal stability of bare and coated PS-NPs was assessed under different conditions of ionic strength and pH.

**Figure 3:**
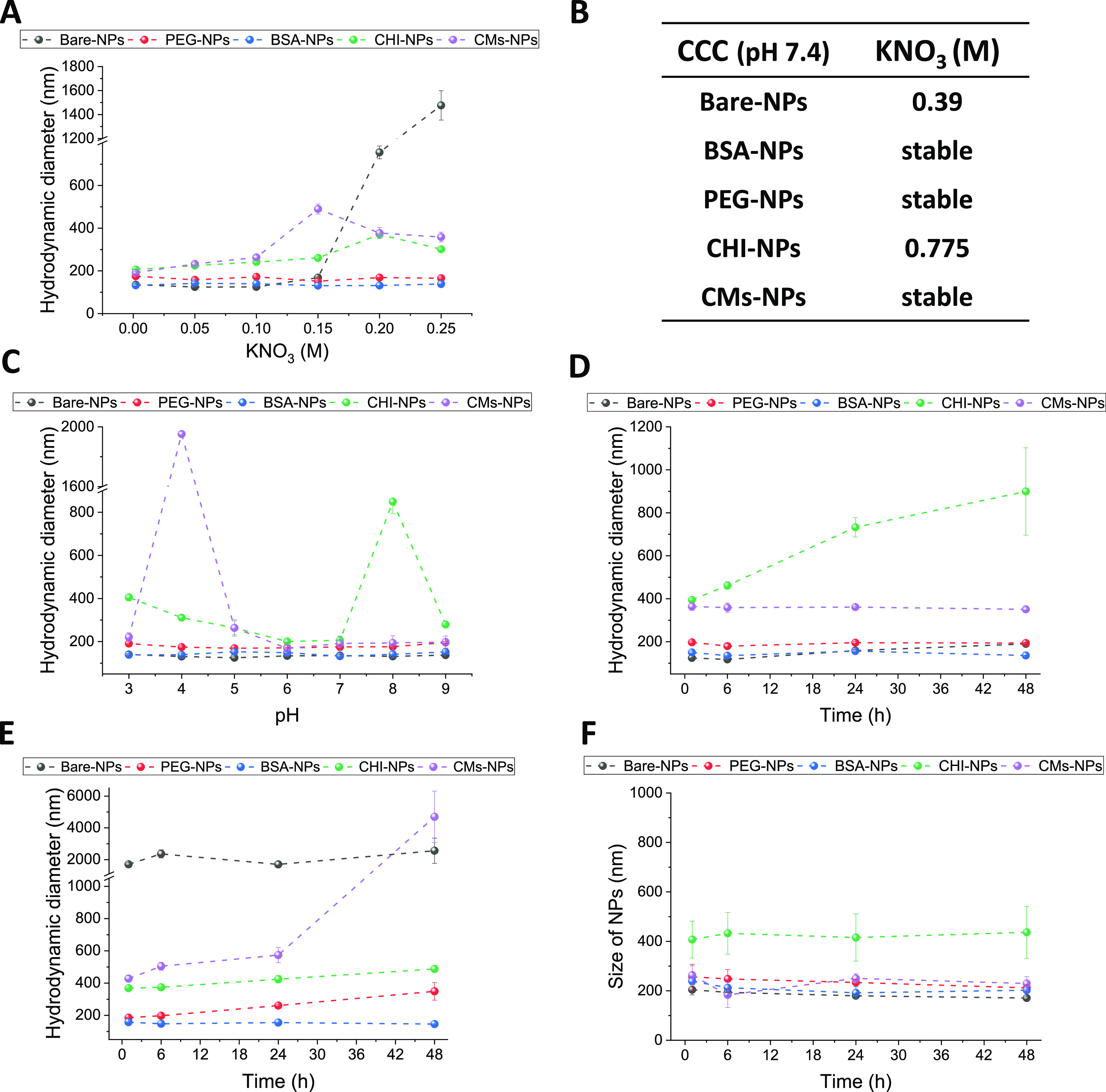
(A) Size of the prepared NPs for increasing concentrations of KNO_3_ (*n* = 3; mean ± SD). (B) CCC data (mM), at pH 7.4, were obtained using KNO_3_ as the aggregating salt. (C) Size of NPs from pH 3 to 11 (*n* = 3; mean ± SD). Size of NPs in (D) PBS (E) DMEM and (F) DMEM with 10% FBS (*n* = 3; mean ± SD).

The effect of the ionic strength of the medium on the average-*D*_H_ was analyzed by varying the concentrations of KNO_3_ at a stable pH of 7.4 (Figure 3A). It was observed that coated NPs showed enhanced colloidal stability in the ionic strength range studied compared to bare NPs. Size plots confirmed this behavior (Figure S3). However, it should be mentioned that a few aggregates started to appear in CHI- and CMs-NP samples at higher ionic strength, as evidenced by the presence of a hump in the size plots (Figure S3). This caused the average size to increase in Figure 3A. Zeta potential of the prepared NPs with increasing concentrations of ionic strength is presented in Figure S4A. Furthermore, we tested the CCC of the prepared nanosystems. By measuring the CCC, we gain insights into the conditions under which particles initiate coagulation and form aggregates, which is essential for understanding the stability of colloidal systems.^34^ KNO_3_ was employed as the aggregating salt (Figure 3B). In consonance with the previous assay, bare-NPs are the least-stable system and undergoes aggregation at 0.35 M KNO_3_ (Figure S4B). CCC could also be determined for CHI-NPs under a higher ionic strength condition of 0.79 M (Figure S4C). PEG- BSA- and CMs-NPs remained stable under the tested conditions. Therefore, coated NPs showed an enhanced colloidal stability against ionic strength compared to bare NPs. The main mechanisms that explain the increased stability of coated systems when electrostatic repulsion stabilization is reduced due to charge screening are the steric impediments and forces provided by the different coatings. For example, it is well described that PEG coating forms a layer on the surface of NPs, creating a steric barrier that prevents aggregation.^6^ In the case of BSA- and CMs-NPs, hydrophilic proteins also contribute to stabilization through hydration forces. These forces arise due to the interaction between the charged or polar groups on the surface and the water molecules in the surrounding medium, resulting in a hydration shell around the NPs that creates a repulsive force between the NPs. PEG coating provides stability to NPs through steric repulsion, whereas hydration forces are also present in protein-based coatings.^35^ Overall, the inclusion of a coating on the surface of NPs clearly provides a higher colloidal stability.

Similarly, we checked the effect of pH on *D*_H_ of the prepared NPs (Figure 3C). All systems remained stable in the tested pH range, except for the CHI-NPs and CMs-NPs that aggregated at pH 8 and 4, respectively (Figure S5). As observed in Figure 2B, the IEP of CHI-NPs and CMs-NPs seems to be found near those pH values, which could explain the observed destabilization.

When introduced into biological environments, NPs are exposed to a wide array of forces arising from the lipids, electrolytes, and proteins present in the media, leading to substantial alterations in their behavior. Furthermore, it is important to highlight that significant variability exists in how NPs behave in complex media, and this variability is strongly associated with the material and surface characteristics of each nanosystem.^36^ Consequently, it is necessary to thoroughly investigate how these systems react within these complex media. In this context, we studied the behavior of the prepared nanosystems under various conditions including PBS, protein-free culture media (DMEM), and complete culture media (DMEM with 10% FBS).

In PBS (0.154 M and pH 7.4), NPs remained colloidally stable at the time points studied, except for the CHI-NPs that showed a tendency to aggregate (Figure 3D). This behavior is evident in the size plots (Figure SX), where CHI-NPs exhibit aggregation over time. Interestingly, a few aggregates are also observed in CM-NP samples from the beginning, causing an initial increase in the average size. However, over time, the samples remain stable. These aggregates do not significantly impact the overall stability of the CMs-NPs over time as the average size of the sample and the size peak of CMs-NPs remain constant. This phenomenon is likely related to the ionic strength, as observed in Figure 3A, which leads to the generation of a few aggregates in the CM-NP sample. CMs-NPs are a relatively new area of study, and further research is needed for a comprehensive understanding. As demonstrated in this work, there are still discussion about the isolation of CMs. Additionally, the coating process raises concerns as the coverage and coating integrity of CM coatings can significantly affect system performance.^21^ It is possible that not all bare NPs in our samples are fully coated, and this incomplete coating may contribute to aggregation at higher ionic strengths. Nonetheless, it is essential to emphasize that despite the formation of these few aggregates, the system remains kinetically stable in PBS, with no significant changes in size from the first to the last time point.

The sizes of the prepared NPs in DMEM culture media are presented in Figures 3E and S7. Notably, bare-NPs exhibit aggregation from the first time point, while CMs-NPs gradually increase in size over time, eventually reaching full aggregation at 48 h. CHI- and PEG-NPs also show an increase in size over time, whereas BSA-NPs remain stable. Interestingly, CHI-NPs, which were not stable in PBS, displayed a better colloidal stability in protein-free media. The differing behavior of the NPs between PBS and DMEM can be attributed to the distinct compounds and reducing environment of the culture media.^37^

The behavior of NPs in complete culture media is shown in Figure 3F. Measuring the size of NPs with DLS in biological media is challenging due to the polydispersity caused by biological components.^37^ Therefore, we employed the size corresponding to the NP population peak rather than the average size of the sample. Interestingly, bare and CMs-NPs, which were unstable in protein-free media, demonstrated stability over time in complete culture media. This observation aligns with the understanding that serum can decrease aggregation because proteins are adsorbed onto the surface of the particles, providing additional stability to nanosystems 39. However, this is not a general rule as cases of NPs that are stable in protein-free media and unstable in serum-containing media have also been reported.^36^ For example, CHI-NPs, which were stable in DMEM, exhibited aggregation in complete DMEM, as is evident in the size plots (Figure S8). In this context, we concluded that CHI-NPs showed the lowest colloidal stability in biological media and emphasized the significance of conducting these types of experiments.

### 3.4 AFM Analysis

AFM and nanomechanical analysis are becoming increasingly important in different fields, such as cancer and developmental biology. The mechanical properties of NPs have a significant impact on various biological aspects, including blood circulation, biodistribution, tumor targeting, and internalization by tumor cells.^38^ For example, stiffness plays a crucial role in NP biodistribution. Softer NPs, which can deform in response to macrophage-induced forces, are less susceptible to macrophage sequestration.^38^ As a result, softer NPs tend to remain in the vasculature for more extended periods compared to their stiffer counterparts. Moreover, the elasticity and deformability of NPs allow them to navigate through small pores while maintaining their structural integrity, contributing to their extended circulation time. In contrast, NPs with limited deformability tend to accumulate in the spleen, resulting in a shorter circulation period.^39^ Furthermore, elastic and adhesive NPs exhibit enhanced cell interactions due to their ability to deform into a flattened configuration, facilitating stronger adhesion.^40^

In this work, AFM was employed to determine the nanomechanical properties of the nanosystems. AFM was operated in the force PinPoint mode to perform distribution map measurements of material components and extract information regarding the stiffness, adhesion, and Young modulus of the NPs (Table 1). In AFM, both stiffness and elastic modulus serve as measures of a sample’s resistance to deformation. Stiffness, an extrinsic property of the material, is calculated as the ratio between the applied force and the resulting deformation of the sample. On the other hand, Young’s modulus or modulus of elasticity is an intrinsic property defined as the ratio of stress to strain. Adhesion represents the attraction between the atoms of a surface and the AFM probe. This force can be determined by measuring the degree of distortion in a cantilever as the tip is retracted from the surface. It should be noted that, with the AFM approach used (as described in the Supporting Information), absolute values cannot be obtained. Consequently, the values presented in Table 1 are relative. However, they can still be effectively compared among the tested NPs.

**Table 1:** Table Presenting the Nanomechanical Properties of the Prepared NPs Obtained for the Analysis of the Measured F-D Curves (*n* = 30; Mean ± SD)

NP	stiffness (N/m)	elastic modulus (GPa)	adhesion (nN)
bare-NPs	5.5 ± 0.3	8.2 ± 0.3	26.9 ± 6.7
PEG-NPs	5.3 ± 0.1	5.9 ± 0.2*	7.7 ± 1.3*
BSA-NPs	5.6 ± 0.3	7.9 ± 0.2	14.6 ± 3.4*
CHI-NPs	5.1 ± 0.5	7.9 ± 0.4	28.1 ± 5.0
CMs-NPs	5.6 ± 0.6	7.8 ± 0.2	28.6 ± 4.9

**Figure 4:**
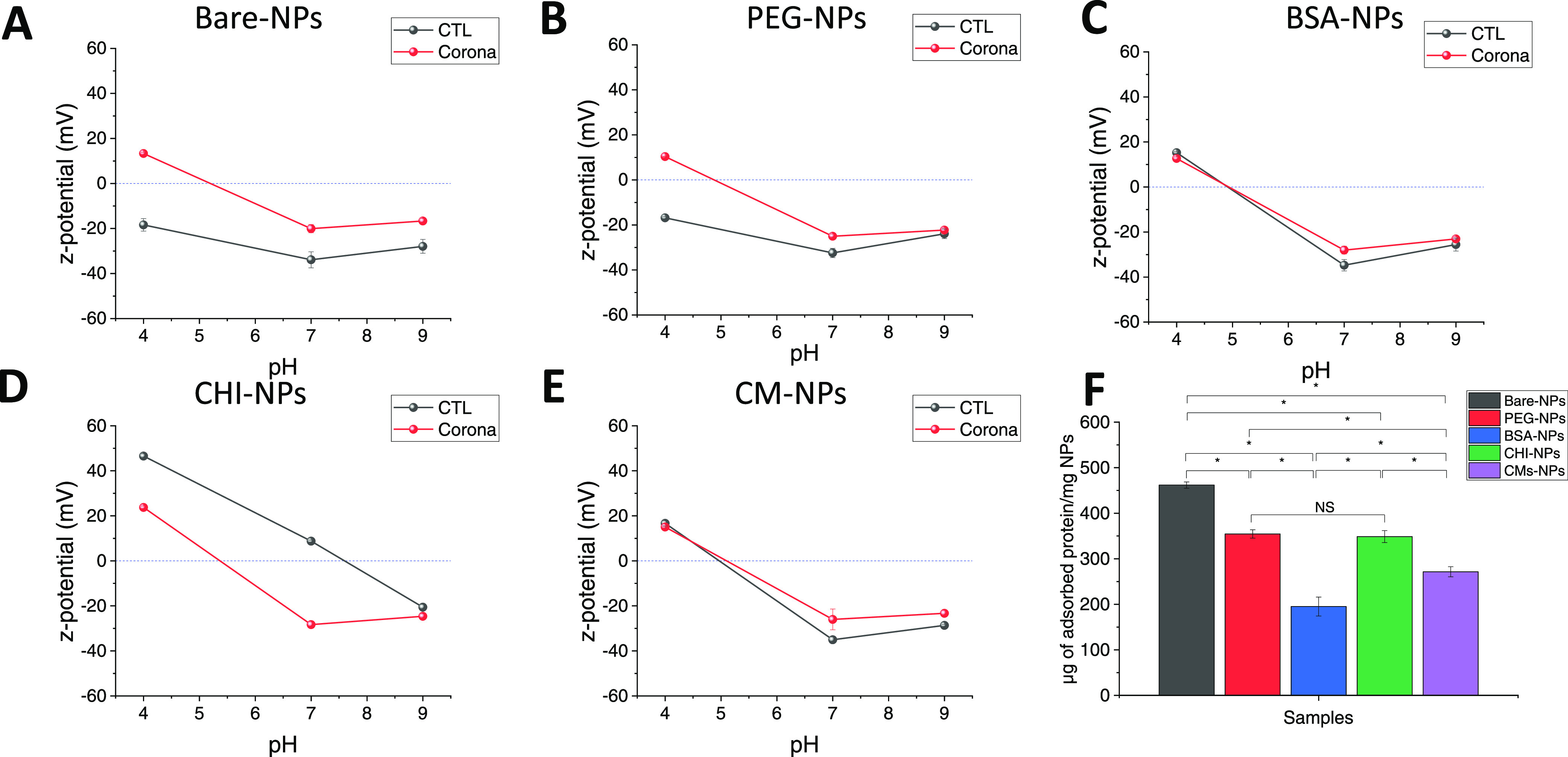
Z-potential (A–F) of the prepared NPs before and after their incubation with complete DMEM and NP complex isolation measured at pH 4, 7, and 9 (*n* = 3; mean ± SD). (F) Analysis of the BCA protein quantification assay. The displayed concentration is corrected to the naked NPs (*n* = 3; mean ± SD). All samples were found different from each other, except for PEG- and CHI-NPs (*p* < 0.05, ANOVA one-way).

Significant differences (*p* < 0.05, ANOVA one-way) were found in the adhesion force and elastic modulus. For instance, NPs coated with BSA and PEG showed a decrease on both the adhesion and Young modulus. No differences were observed on the stiffness of the particles. Unlike the stiffness, which depends mainly on the mechanical characteristics of the biomaterial (in this case, the polystyrene from the core of the NPs), the adhesion and Young modulus are dependent on the surface chemical properties as the roughness and hydrophobicity.^41^ Therefore, the stiffness values of all particles remained almost equal since they possess the same core material. The lower elastic modulus and adhesion obtained for PEG-NPs could indicate that the PEG coating becomes more deformed than the core material in contact with the AFM tip, giving a higher elasticity to the overall particle. For instance, bare NPs have a slightly larger elastic modulus, meaning that the NPs with no coating are more resistant to deformation. Furthermore, adhesion decreases when particles are covered with PEG and, to a lesser extent, BSA (Figure 4K). In this sense, it can be concluded that PEG and BSA-NPs are softer than the rest.

These results support the idea that adhesion and the elastic modulus depend not only on the core material of particles but also on their surface. We observed that BSA- and, especially, PEG-NPs exhibited softer behavior than the other systems. As established, softer NPs generally have longer circulation times and can evade macrophage uptake. In this sense, coatings, such as BSA and PEG, which provide NPs with a softer surface, can offer these advantages. In contrast, CHI and CMs do not significantly alter these nanomechanical properties. Therefore, coating NPs with different shells can indeed modify the nanomechanical properties of NPs which is crucial to understanding the behavior of these systems in the biomedical field.

### 3.5 Protein Corona

When NPs enter a complex physiological medium, the surrounding biomolecules are adsorbed onto their surface, forming a biomolecular corona. This corona creates a new identity for the NPs, which influences their properties and interactions with biological components since it imparts new recognition motifs that may trigger unintended biological mechanisms. To study the protein corona, we incubated the prepared NPs with DMEM culture media supplemented with 10% FBS. Changes in surface potential at different pH values after incubation reflected the presence of proteins adsorbed onto the NP surfaces, modifying their original surface charge density (Figure 4A–E). NP–protein complexes showed protein-type behavior, changing their z-potential from positive at acidic pH to negative at higher pH levels. Clear differences appeared, depending on the nature of the NP shell. This was especially evident for CHI-NPs, as the surface charge at pH 7 and 9 changed from positive to negative (Figure 4D). BSA-NPs and CM-NPs remained practically unchanged (Figure 4C,E), whereas the surface potentials of bare-NPs and PEG-NPs reduced at pH 7 and 9 and changed to positive values under acidic conditions (Figure 4A,B). Corona–NP complexes also showed higher hydrodynamic sizes compared to naked NPs (Figure S9), which can be attributed to the now present protein corona shell around the NPs. However, CHI-NPs exhibited a particularly noticeable increase in size, which could indicate aggregation of the system. As mentioned previously, CHI-NPs have the lowest colloidal stability compared to the other systems. It should also be considered that the centrifugation process employed to isolate the corona-CHI-NPs may have had a negative effect in their stability. Interestingly, although naked CMs-NPs were aggregated at pH 4, their, respective, corona-NP complexes did not suffer this drastic destabilization, meaning that the protein corona can have a stabilization effect on the NPs.

Furthermore, we used the BCA method to estimate the amount of protein adsorbed on the prepared NPs.^42^ Naked barley, PEG-, BSA-, CHI-, and CMs-NPs were used as controls. In comparison to bare NPs, all coated NPs exhibited a significant reduction in the number of adsorbed proteins (Figure 4F). Specifically, PEG and CHI coatings resulted in a 20% reduction in adsorbed proteins, while CM-NPs and BSA-NPs achieved a 40 and 60% reduction, respectively, when compared to bare NPs (*p* < 0.05, ANOVA one-way). This implies that all coatings effectively prevented the adsorption of proteins from the culture media. As observed, the number of adsorbed proteins is highly dependent on the NP’s surface properties. Previous studies have already highlighted the capacity of PEG and CHI coatings to reduce protein corona formation.^43,44^ It should be noted that the adsorption of proteins on PEG-coated NPs is dependent on the PEG density, achieving a higher reduction with a higher density of grafting.^44^ Protein corona reduction for BSA-coated NPs has previously been described.^45^ Similarly, Rao et al. reported that erythrocyte membrane coating on upconversion NPs (UCNPs) can effectively prevent protein adsorption compared to noncoated UCNPs.^46^

The protein corona plays a crucial role in shaping the pharmacokinetics, biodistribution, and toxicity profiles of NPs. A reduction in the protein corona could theoretically lead to diminished recognition, resulting in favorable outcomes. In this context, both BSA and CM coatings have shown particularly promising results. However, it is not just the quantity of proteins that matter but also their specific composition within the corona. For instance, studies have demonstrated that PEGylated surfaces can reduce clearance by immune cells by selectively adsorbing certain proteins onto their corona.^44^ Nevertheless, it is essential to note that the complete prevention of protein corona formation remains a challenge. The field of protein corona research is continuously evolving, with ongoing debates and investigations into whether specific components within the protein corona can trigger NP internalization.^47^ Therefore, a comprehensive understanding of the protein corona remains indispensable in the design of NPs for biomedical applications. Importantly, this study contributes to the comprehension of reduced protein corona formation in CM-coated NPs, shedding light on their potential advantages in various biomedical contexts.

### 3.6 In Vitro 2D Cellular Uptake

Upon arriving at the disease site, the efficient uptake of NPs is crucial for the effective delivery of drugs into cells. Different coatings can impact the formation of the protein corona, which in turn affects the bionano interface that the cell encounters, ultimately influencing the efficiency of uptake. The effect of the coating on cellular uptake by breast adenocarcinoma MCF-7 cells was evaluated by flow cytometry and confocal microscopy.

**Figure 5:**
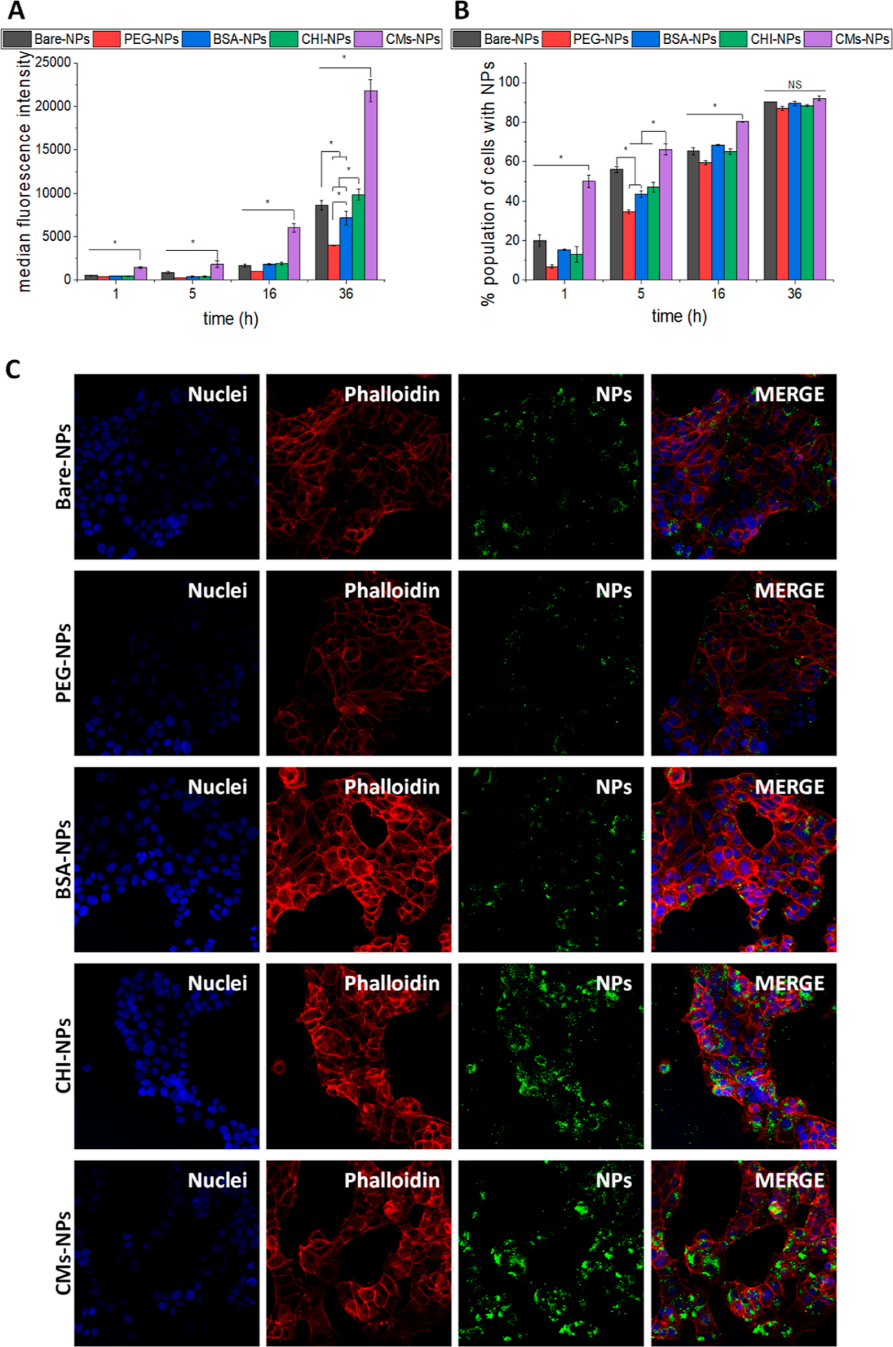
Cellular uptake of the prepared NPs on MCF-7 cells, followed by flow cytometry representing (A) as the mean fluorescence intensity and (B) as the population of cells with NPs. Statistically significant differences between cells incubated with the prepared NPs for the same time point are highlighted with “*”. ANOVA with Tukey mean comparison test (*p* < 0.05) was employed. (C) Confocal fluorescence microscopy images of MCF-7 breast cancer cells incubated with the prepared NPs. Nuclei were stained with Hoechst (blue) and F-actin with Alexa Fluor 647 phalloidin (red). Fluorescent NPs (FITC) appear in green.

**Figure 6:**
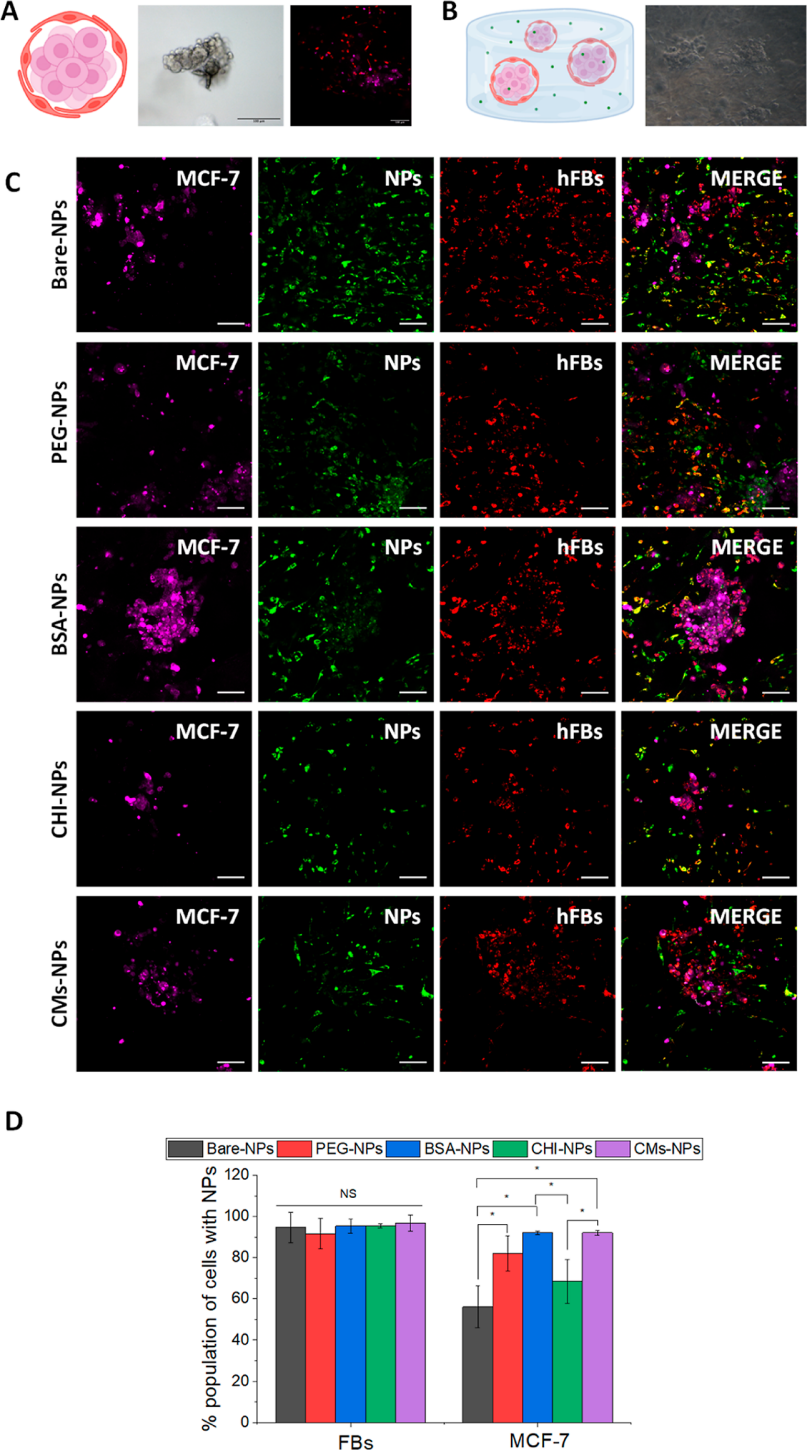
(A) Schematic image, microscopic, and confocal microscopic representative images of multicellular spheroid of MCF-7 cells stained with CellTracker Deep Red (violet) and FBs stained with CellTracker Red (red). (B) Schematic image and microscopic representative image of spheroids embedded with the collagen type-I hydrogel after 48 h of culture. (C) Confocal representative images of spheroids embedded with the collagen type-I hydrogel after treatment with NPs. MCF-7 cells were stained with CellTracker Deep Red (violet) and FBs stained with CellTracker Red (red). Fluorescent NPs (FITC) appear in green. Scale bar: 100 μm. (D) Cellular uptake of the prepared NPs on MCF-7 and FB cells followed by confocal microscopy. Colocalization analysis was performed with Software ImageJ (*n* = 3). Statistically significant differences between cells incubated with the prepared NPs are highlighted with “*” (*p* < 0.05).

For flow cytometry, cells were incubated with 10 μg/mL of FITC-containing NPs (1.8 × 10^5^ NPs/cell) for 1, 5, 16, and 36 h. Flow cytometry results showed that all of the NPs entered the cells even after short incubation periods. Different performances of the prepared NPs were observed (Figure 6A,B). CM-NPs showed significantly higher uptake by cells (*p* < 0.05) for all time points tested compared to the other systems (Figure 5A). At longer incubation times (36 h), different behaviors between the prepared NPs were observed. For instance, PEG and BSA coating reduced the uptake of bare NPs (*p* < 0.05), whereas no differences were found between CHI- and bare NPs. BSA-, PEG-, and CHI-NPs also showed significant differences between them (Figure 6A). In addition, CMs-NPs were the fastest to enter cells, as evidenced by the higher percentage of cells with internalized NPs at short times (Figure 5B). At longer incubation times, all cells eventually contained NPs, but there were significant differences in the quantity of internalized NPs, as previously noted. For confocal microscopy, cells were incubated with 10 μg/mL of each NPs for 36 h. Confocal images showed that NPs were internalized in the cells and distributed within the entire cytoplasm surrounding the cell nucleus (Figure 5B). Consistent with the flow cytometry results, differences in fluorescence intensity were also observed.

Surface charge, hydrophobicity, and functionality play key roles in directing the NP-cell interaction.^47^ Regarding the reduction of internalization of PEG- and BSA- compared to bare NPs, it is stated that coatings which prevent protein adsorption and unspecific binding could also make interactions with the CM and cell receptors more difficult, leading to a lower internalization.^48^ On the other hand, although the chitosan coating also reduces the formation of protein corona, no differences were found between the performance of CHI- and bare NPs. The different surface charges could explain this different behavior. For instance, positively charged NPs showed a more favorable interaction with CMs than negatively charged NPs owing to the electrostatic interactions established between the positively charged surface of the nanosystem and the negatively charged CM, which can give rise to a membrane wrapping phenomena^49^ Interestingly, although CM-NPs also prevent protein binding, they showed a significantly higher internalization rate (>2×). The isolated CMs are composed of a mixture of lipids, proteins, and carbohydrates which are responsible for providing the interfacing functionalities of the CMs. The presence of these markers on the surface of the CMs-NPs has shown to change NP biointerfacing and increase their internalization.^14^ These results suggest that CMs-NPs are capable both of reducing the in vitro formation of protein corona while increasing the in vitro cellular uptake on MCF-7 breast cancer cells.

### 3.7 Uptake in a 3D Tumor Model

Conventional cell monolayer culture approaches have limitations in accurately representing the native tumor context and the behavior of cells in the tumor microenvironment. The lack of a 3D niche and stromal component significantly alters cell morphology, exposed surface area, and cellular signals and interactions with their environment. All these aspects affect in vitro responses of different antitumor therapies, including the transport, penetration, and uptake of drugs and NPs by tumor cells.^50^ It has been extensively demonstrated how the toxic effects of NPs are significantly reduced in cultured 3D models, such as cell spheroids, compared to monolayer culture data.^51^ Furthermore, ECM and stromal cells can alter the accessibility of NPs to the tumor and their internalization in tumor cells as they generate a physical obstacle and trigger biological changes in the tumor that can be a limiting factor in the efficacy of these treatments.^52^ These effects can be elucidated in vitro using three-dimensional models encapsulated in hydrogels, which more closely replicate the in vivo cellular uptake of NPs.^50,52^ Collagen type I was employed to generate an ECM-derived hydrogel as it is the most abundant structural protein in breast cancer tissues and plays a fundamental role in tumor progression and drug resistance.^53^ The behavior of the prepared NPs was analyzed in multicellular spheroids of MCF-7 and FBs embedded in a collagen type-I hydrogel to study their effect in a biomimetic environment comprising the ECM. To discern between tumoral and nontumoral cells, MCF-7 cells were previously stained with CellTracker Deep Red (Figure 6A, marked in violet) and FBs with CellTracker Red (Figure 6A, marked in red), assembled in heterogeneous spheroids. The spheroids were cultured within the hydrogel to allow the cells to adapt to the environment, and after 48 h, it was observed that the cells colonized the hydrogel, and FBs adopted their characteristic spindle-shaped morphology (Figure 6B).

Colocalization analysis of the NPs and cells showed that the NPs with different coatings were able to penetrate through the collagen gel and permeate almost all stromal cells with no significant differences between coated NPs and bare NPs. In native tumor niches, both FBs and ECM exert a chemoprotective effect on tumor cells through different strategies, such as providing a physical barrier around them to prevent drug penetration.^53^ Therefore, it is essential that new therapeutic candidates can bypass these mechanisms and reach the targeted cells. In accordance with this, all NPs reached the tumor cells, but significant differences in their behavior were observed. PEG-, BSA-, and CMs-NPs were significantly more internalized in the tumor population than bare NPs (*p* < 0.05). No significant differences were observed between these three nanosystems in which more than 80% of the tumor population was affected in all cases (Figure 6D). Interestingly, although PEG- and BSA-NPs showed lower internalization in MCF-7 cells in the in vitro 2D uptake compared to bare NPs, higher internalization in the 3D model was observed. As stated, 2D culture systems lack the 3D architecture and complexity of the in vivo microenvironment, which can lead to inaccurate results. In contrast, 3D systems more accurately mimic the physiological conditions of tissues and organs, allowing for a better understanding of NPs’ uptake and distribution within cells. The difference in the uptake of CHI-NPs with respect to the 2D uptake assay may be attributable to the fact that CHI can interact with type-I collagen due to its positive surface charge,^54^ and that, as previously described, CHI-NPs are the less-colloidal stable system, decreasing their availability in the environment. From this experiment, it can be concluded that coating NPs with PEG, BSA, or CMs allows them to reach tumor cells even in protective environments, whereas noncoated NPs do not achieve to surpass this biological barrier.

### 3.8 Cytokine Release

To test the ability of the prepared NPs to activate an inflammatory response, THP-1 monocyte cells were incubated with the prepared NPs, and the secretion of the pro-inflammatory cytokines IL-1, IL-6, IL-8, GM-CSF, MCAF, and TNF-α was evaluated using an ELISA multiplex. Pro-inflammatory cytokines such as IL-1, IL-6, and TNF-α are important for the initiation of inflammation and activation of innate and adaptive immune cells. In particular, both IL-1 and IL-6 bind to macrophages and T cells, activating the JAKs-STAT and NF-κB pathways. IL-6 also induces the differentiation of B cells for production of antibodies.^55^ During the acute inflammatory response, TNF-α possesses multiple roles, such as the activation of inflammatory cytokines, neutrophils, and lymphocytes and the increase in the permeability of the vascular endothelium.^56^ Similar to IL-6, IL-8 is a chemotactic factor that attracts T-cells, neutrophils, and basophils.^57^ GM-CSF is an extracellular polyprotein that functions as an immune modulator, serving as a potent immune adjuvant that induces long-lasting antitumor immunity, and MCAF causes the degranulation of basophils and mast cells and increases the activity of monocytes and macrophages.^58,59^

**Figure 7:**
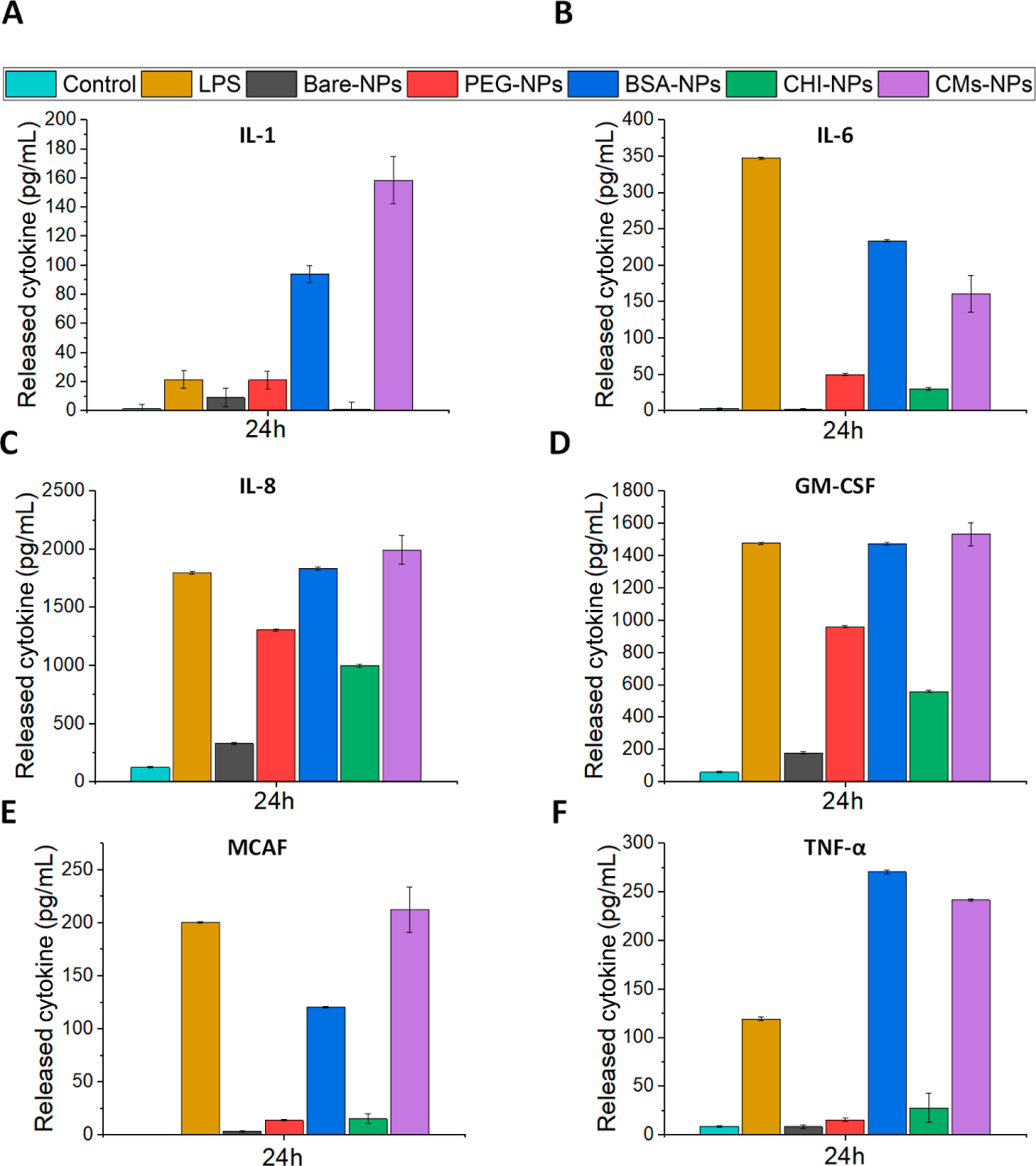
Cytokine release was assessed by multiplex ELISA on THP-1 cells. Cells were incubated with the prepared NP nanocapsules at a dose of 10 μg/mL or left untreated. Lipopolysaccharides (LPS 1 μg/mL) were used as a positive control. After 24 h, the supernatants were collected and analyzed. (A–F) IL-1, IL-6, IL-8, GM-CSF, MCAF, and TNF-α were analyzed.

The impact of the differently functionalized NPs on THP-1 cells was confirmed by testing the release of the aforementioned cytokines (Figure 7). An endotoxin assay (Thermo Scientific Pierce Chromogenic Endotoxin Quant Kit) was performed on the NP suspensions before they were administered to the cells, yielding negative results. BSA- and CMs-NPs generated a higher release of cytokines compared to that of the rest of the nanosystems. For instance, the incubation with these two NPs produced a higher cytokine release than the positive control for IL-1 and TNF-α, whereas for IL-8 and GM-CSF, the cytokine concentration was similar to that observed in the incubation with LPS. Furthermore, CMs-NPs produced a higher release of IL-1 and MCAF than BSA-NPs, while the opposite was found for IL-6. For IL-1, IL-8, and TNF-α, a similar release was found for BSA- and CMs-NPs. Additionally, compared to cells incubated with PBS, the concentration of IL-6, IL-8, and GM-CSF was increased after the incubation with PEG- and CHI-NPs, whereas no significant variations of IL-1, MCAF, and TNF-α were observed for these nanosystems. Bare NPs produced a slight increase in the release of IL-8 and GM-CSF compared to that of the control.

Cytokine analysis confirmed that the prepared nanosystems could stimulate monocytes to produce proinflammatory cytokines, especially BSA- and CMs-NPs. The role of inflammation in the context of nanotherapy is multifaceted and has significant implications. Traditionally perceived as a negative aspect, inflammation, when strategically harnessed, can offer unique advantages, especially for tumor treatment. For patients exhibiting a diminished immune response, leveraging inflammation as an adjuvant becomes an intriguing prospect. Acute inflammation contributes to cancer cell death by inducing an antitumor immune response.^60^ In this sense, CM-coated NPs are promising for tumor immunotherapy as they retain interesting antigens on their surface, directly enhancing the efficacy of immunotherapy.^61^ Deng et al. treated macrophages with NPs coated with membranes from human natural killer cells and observed an increase in TNF-α, IL-6, and IL-12 production and a decrease in the M2-macrophage-related cytokine IL-10, compared to noncoated NPs.^62^ Similarly, the effect of BSA-NPs on the cytokine profile of THP-1 cells was previously established by Cochran and Finch-Arietta, who showed that BSA-coated beads were a potent stimulus for IL-1 production, comparable to the maximal dose of LPS.^63^ Zhao et al. also observed enhanced secretion of IL-1b and TNF-a by microglia cells when treated with BSA.^64^ CHI and PEG coating has also been shown to increase the release of pro-inflammatory cytokines.^65,66^ In line with our results, Prietl et al. observed a slight increase in IL-6 and IL-8 release upon incubation of THP-1 cells with carboxyl polystyrene particles for 24 h at 10, 20, and 50 μg/mL.^67^ Taken together, these results indicate that coated NPs, especially BSA- and CMs-NPs, can induce an acute inflammatory process by prompting monocytes to secrete proinflammatory cytokines, offering potential applications in the context of nanotherapy and immunotherapy. However, while activating the immune system as a coadjuvant offers intriguing possibilities, there are still severe safety concerns due to the complex interplay of inflammation. Furthermore, it is crucial to note that the results from these in vitro experiments may not directly translate to an in vivo setting. Consequently, further studies are required to thoroughly assess the implications of these findings.

## 4 Conclusions

The obtained results allowed us to study both the colloidal and physicochemical characteristics of the prepared NPs as well as their interaction and performance in a biological environment. From a colloidal point of view, coatings provide steric stabilization to NPs that keeps them stable. CHI coating resulted in the least colloidally stable nanosystems, mainly because the isoelectric point of chitosan is close to the pH of biological systems. Herein, we demonstrate that AFM is a suitable technique for studying the nanomechanical properties of nanosystems. Furthermore, it was determined that adhesion and elastic modulus of NPs were clearly dependent on the surface and, therefore, the coating of the system. Efficient biointerfacing of NPs with biological environments is crucial to the production of effective nanosystems. Protein corona formation is one of the first obstacles that NPs encounter when they enter the bloodstream. A reduction of protein adsorption in coated NPs was shown, especially evident for BSA- and CM-coated NPs. CMs-NPs showed a 2× higher cellular uptake rate compared to the rest of the systems. 3D cell culture models are becoming increasingly important for studying NP internalization and their potential applications as drug delivery systems. In this sense, uptake of the prepared NPs in multicellular spheroids of MCF-7 and FBs embedded in a collagen type-I hydrogel showed that PEG, BSA, and CM coatings allowed NPs to reach tumor cells even in protective environments compared to bare NPs. Cancer cells have evolved a series of mechanisms to achieve immune escaping. NPs with the ability to activate an inflammatory response can alleviate the tumor immunosuppressive environment, thus achieving better antitumoral effects. The results presented here showed that coated NPs, especially BSA-NPs and CMs-NPs, induced THP-1 monocytes to release proinflammatory cytokines, thereby inducing an acute inflammation process and T cell responses. Taken together, these findings indicate that the characteristics of the coating have a significant impact on the behavior of nanosystems. Notably, CMs-NPs demonstrate a substantial reduction in protein corona formation and an increase in cellular uptake. CM coating technology has established itself as a promising candidate that holds significant potential for personalized medicine and enhanced therapeutic outcomes. However, it is essential to acknowledge the practical challenges and issues that need to be addressed to fully harness the potential of the CM coating technology.

Primarily, CM coating as a top-down approach entails working with a highly complex material. Furthermore, different extraction protocols for CM are documented in the literature, which, as evaluated in this work, yield CMs of varying purity, and consequently, different behaviors of the CM-NPs should be expected. It is indeed necessary to conduct an exhaustive exploration to optimize extraction methods, considering different cell lines and lysis methods, among other variables. Additionally, the coating process raises concerns. The coverage and coating integrity of the CM coatings can also significantly influence the performance of these systems.

In summary, our research underscores the promise of CM coatings as a compelling approach in the field of nanomedicine, outperforming other commonly employed coatings. While practical challenges must be overcome, our work contributes to a deeper understanding of these technologies and the avenues for further exploration, making them more accessible for clinical applications and personalized medicine.

## Data Availability

The data that support the findings of this study are available from the corresponding author upon reasonable request.

## Supporting Information Available

SDS-PAGE of the prepared BSA-NPs and CMs-NPs, TEM micrographs, size distribution by number of the prepared nanosystems against higher concentration of ionic strength, zeta-potential of the prepared NPs, size distribution by number of the prepared nanosystems as a function of the pH, zeta-potential of the prepared NPs for increasing concentrations of KNO3, size distribution by number of the prepared nanosystems as a function of the pH, size distribution by number of the prepared nanosystems in PBS, size distribution by number of the prepared nanosystems in DMEM, size distribution by number of the prepared nanosystems in DMEM with 10% FBS, size of the prepared NPs before and after their incubation with complete DMEM and NP complex isolation, and calibration curves (PDF)
