# Supplementary material for: Exploring the Impact of Nanoparticle Stealth Coatings in Cancer Models: From PEGylation to Cell Membrane-Coating Nanotechnology
Source: ACS Appl Mater Interfaces. 2023 Dec 30;16(2):2058–74. doi: 10.1021/acsami.3c13948 (PMC10797597; doi:10.1021/acsami.3c13948)
Supplement: Supplementary file 1 — am3c13948_si_001.pdf [file am3c13948_si_001.pdf]

Exploring the Impact of Nanoparticle Stealth Coatings in Cancer Models: From PEGylation to Cell Membrane Coating Nanotechnology

Pablo Graván [a,b,c,d,e,f] , Jesús Peña-Martín [b,c,d,e,f], Julia López de Andrés [b,c,d,e,f], María Pedrosa [a,e], Martín Villegas-Montoya [a,e,g], Francisco Galisteo-González [a*], Juan A. Marchal [b,c,d,e,f,*], Paola Sánchez-Moreno [a,e,*]

[a] Department of Applied Physics, Faculty of Science, University of Granada, 18071 Granada, Spain.

[b] Department of Human Anatomy and Embryology, Faculty of Medicine, University of Granada, 18016 Granada, Spain.

[c] Instituto de Investigación Biosanitaria de Granada (ibs.GRANADA), 18012 Granada, Spain.

[d] Biopathology and Regenerative Medicine Institute (IBIMER), Centre for Biomedical Research (CIBM), University of Granada, 18016 Granada, Spain.

[e] Excellence Research Unit Modelling Nature (MNat), University of Granada, 18016 Granada, Spain.

[f] BioFab i3D - Biofabrication and 3D (bio)printing laboratory, University of Granada, 18100 Granada, Spain.

[g] Faculty of Biology, Calzada de las Américas and University, Ciudad Universitaria, 80040 Culiacán, Sinaloa, Mexico.

* Co-corresponding authors

E-mail: paolasm@ugr.es; jmarchal@ugr.es; galisteo@ugr.es

Methods

AFM analysis

For non-contact mode analysis ACTA cantilevers (K = 40 N m^{-1} and f = 320 kHz) were employed. Images were acquired as 256 × 256 pixels at a scan rate of 0.5 – 0.7 Hz. Subsequently, images were processed and analyzed using Gwyddion Open-Source software. Representative images of samples were obtained by scanning at least 3 different locations on at least 3 different samples of the same nature. For Force Spectroscopy NSC-14 probes were used (K = 5 N m^{-1} and f = 160 kHz) and measurements were performed using the gentle PinPoint mode to acquire reproducible and reliable topography, stiffness, adhesion and elastic modulus maps. This technique combines the horizontal and vertical movement of the tip in an approach-retract manner, and allows to apply forces of only a few nano-Newtons to preserve the sample. The software simultaneously acquires topographical data and the force-distance (F-D) curve at each pixel. In addition, at least 30 force-distance (F-D) curves were obtained in each area, only when the point was in contact with a nanoparticle. The values of stiffness, adhesion and elastic modulus have been obtained based on the study of F-D curves according to Park Systems specifications (https://www.parksystems.com/park-spm-modes/95-force-measurement/244-force-distance-spectroscopy) (https://www.parksystems.com/applications/life-science/cell-biology/12-park-pinpoint-mode-for-cell-biology?highlight=WyJwaW5wb2ludCJd) . Stiffness is given by the slope in the contact region of the F-D curve, adhesion is the minimum force in the curve, and the elastic modulus is calculated by converting the displacement in z to tip indentation and applying the Hertz model to take into account the geometry of the tip.

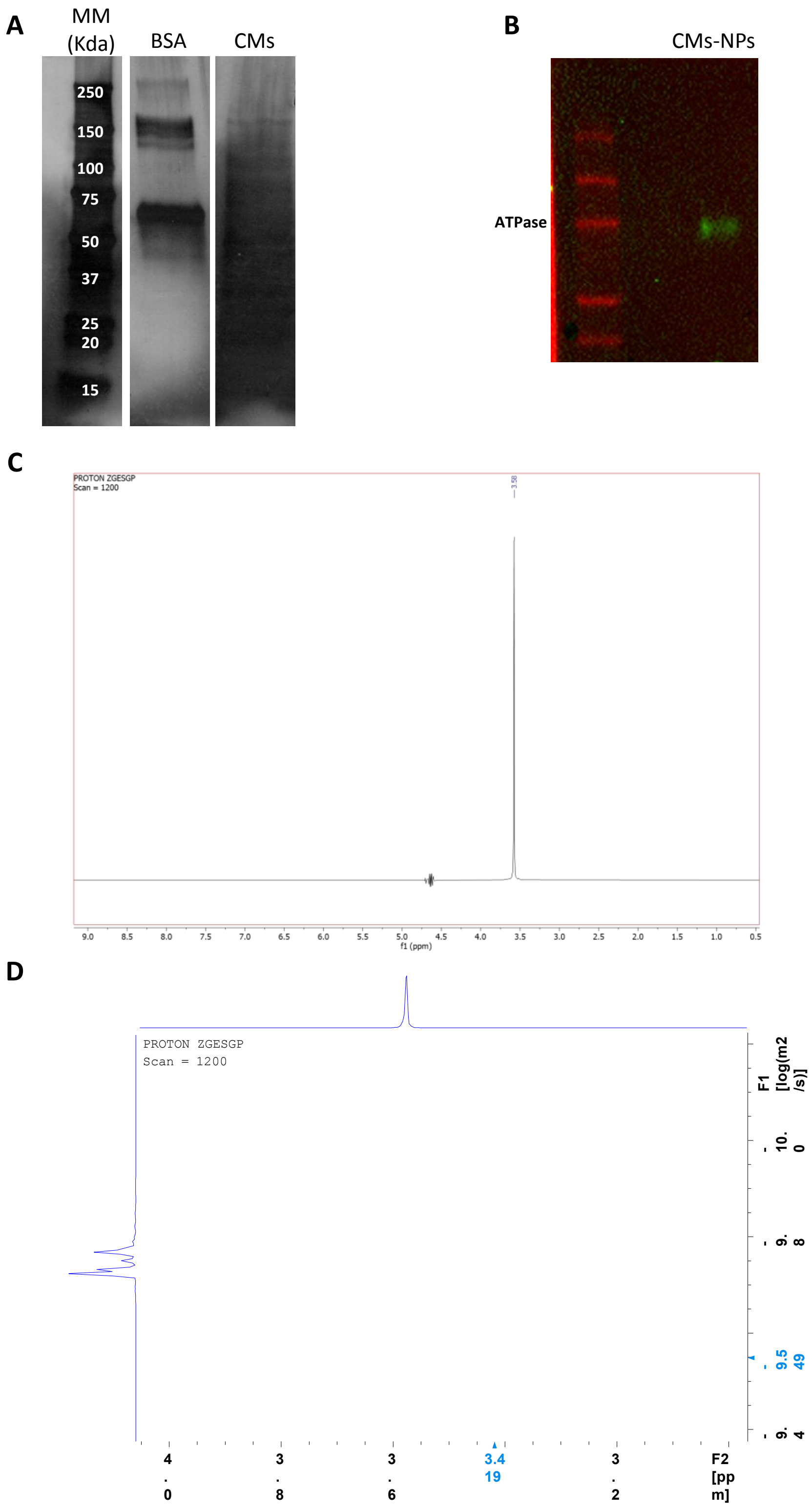

A
MM (Kda)
BSA
CMs
250
150
100
75
50
37
25
20
15
B
CMs-NPs
ATPase
C
PROTON ZGESGP
Scan = 1200
f1 (ppm)
D
PROTON ZGESGP
Scan = 1200
F1 [log(m2/s)]
F2 [ppm]

E

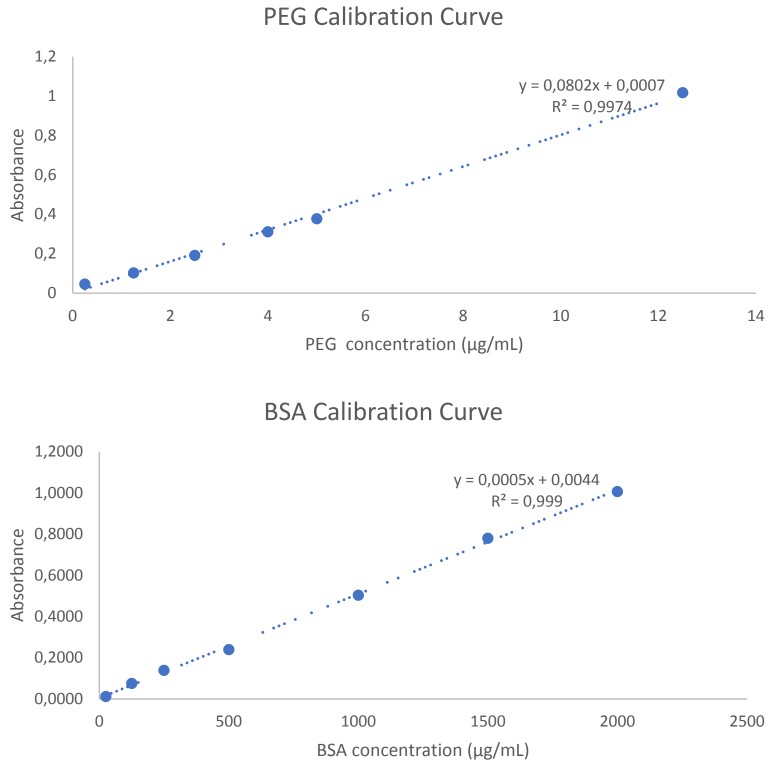

Figure S1. **(A)** SDS-PAGE of the prepared BSA-NPs and CMs-NPs. **(B)** western blot analysis of the prepared CMs-NPs. **(C)** 1H NMR spectrum (water-suppressed) of intact PEG-NPs (24ºC, 500 MHz) and (**D**) Diffusion-ordered NRM spectrum (DOSY) of PEG-NPs (24ºC, 500 MHz). (**E**) Calibration curves for PEG and BSA quantification.

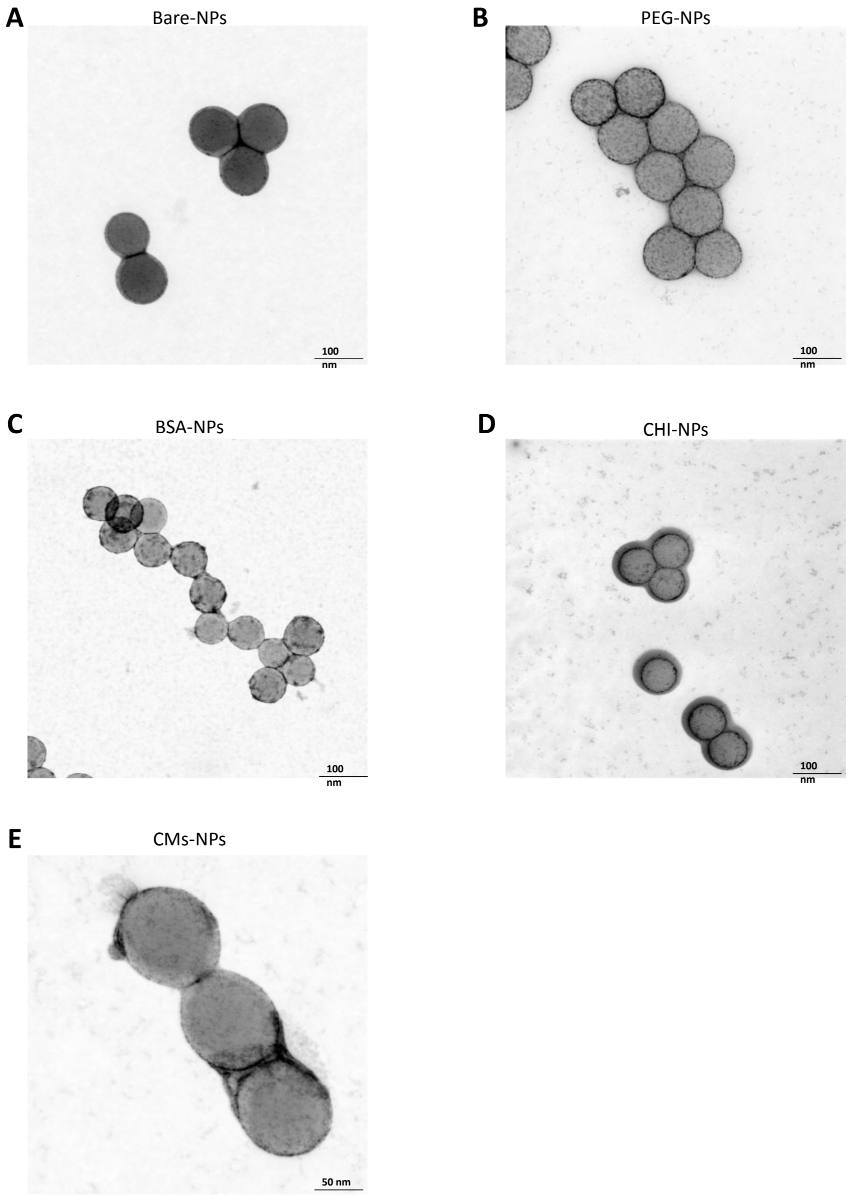

Figure S2. TEM micrographs operated at STEM mode of uncoated **(A)** and coated NPs **(B-E)**, PEG-NPs, BSA-NPs, CHI-NPs and CMs-NPs, respectively.

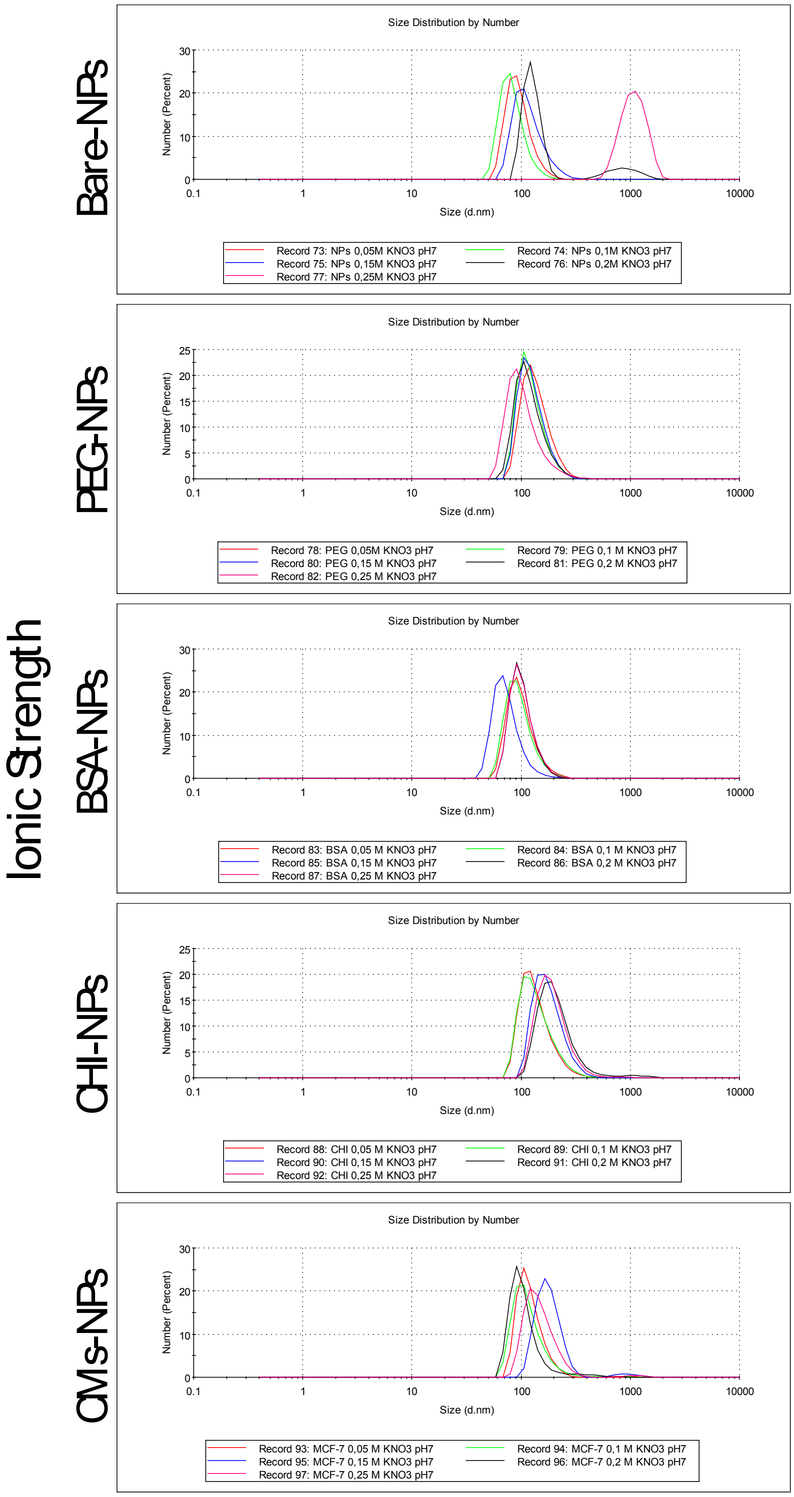

Figure S3. Size distribution by number of the prepared nanosystems against higher concentration of ionic strength.

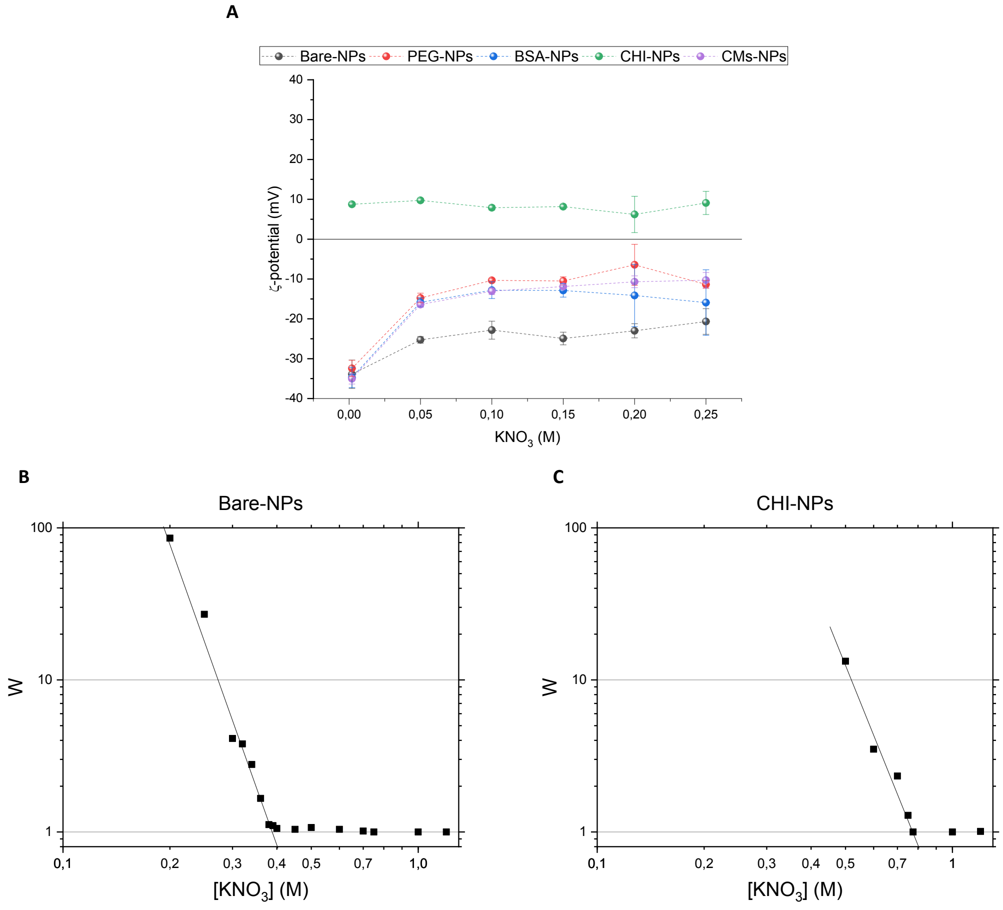

Figure S4. (A) zeta-potential of the prepared NPs for increasing concentrations of KNO_3 (n = 3; mean ± SD). CCC determination by calculating the Fuchs factor (W) of **(B)** Bare-NPs and **(C)** CHI-NPs.

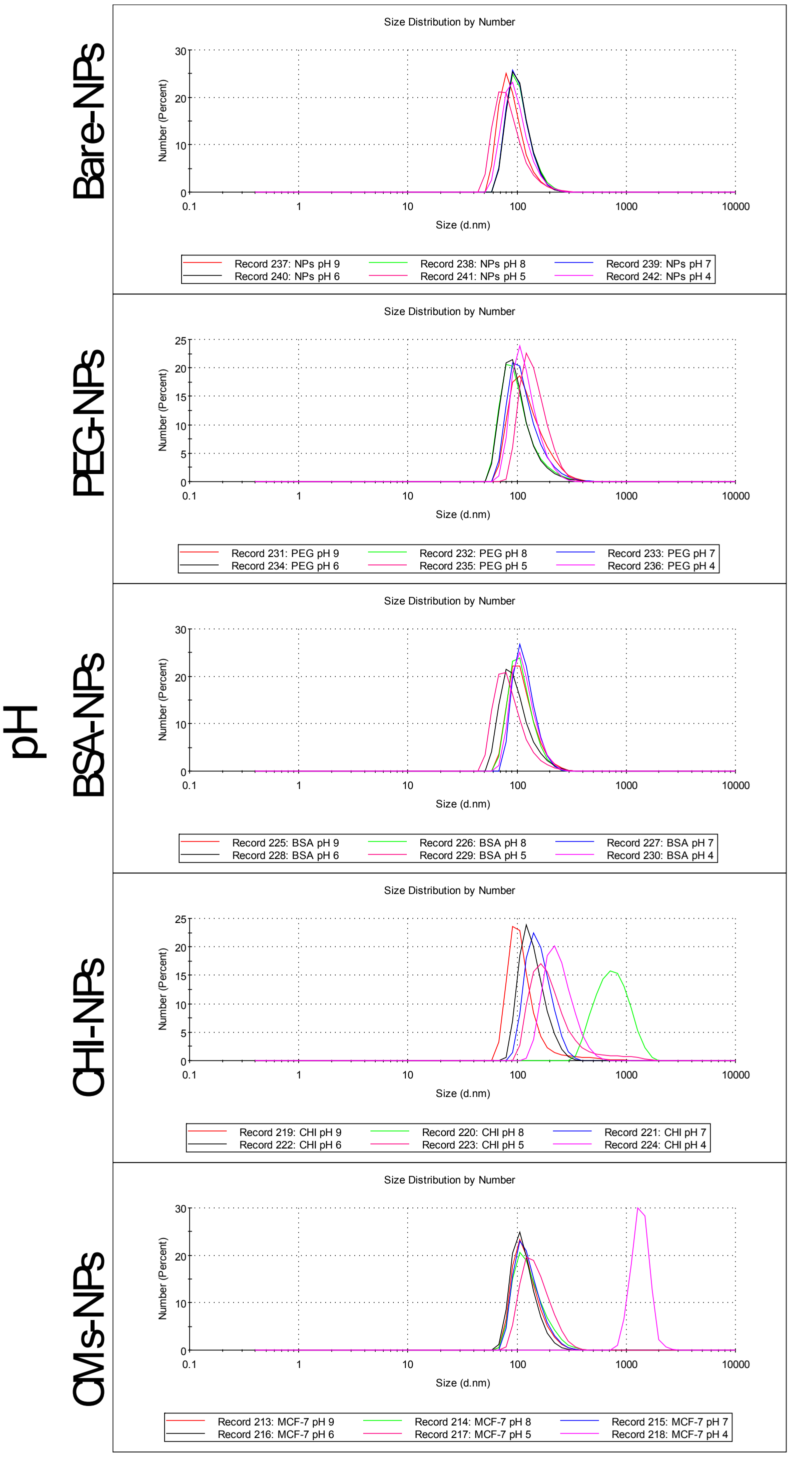

Figure S5. Size distribution by number of the prepared nanosystems as function of the pH.

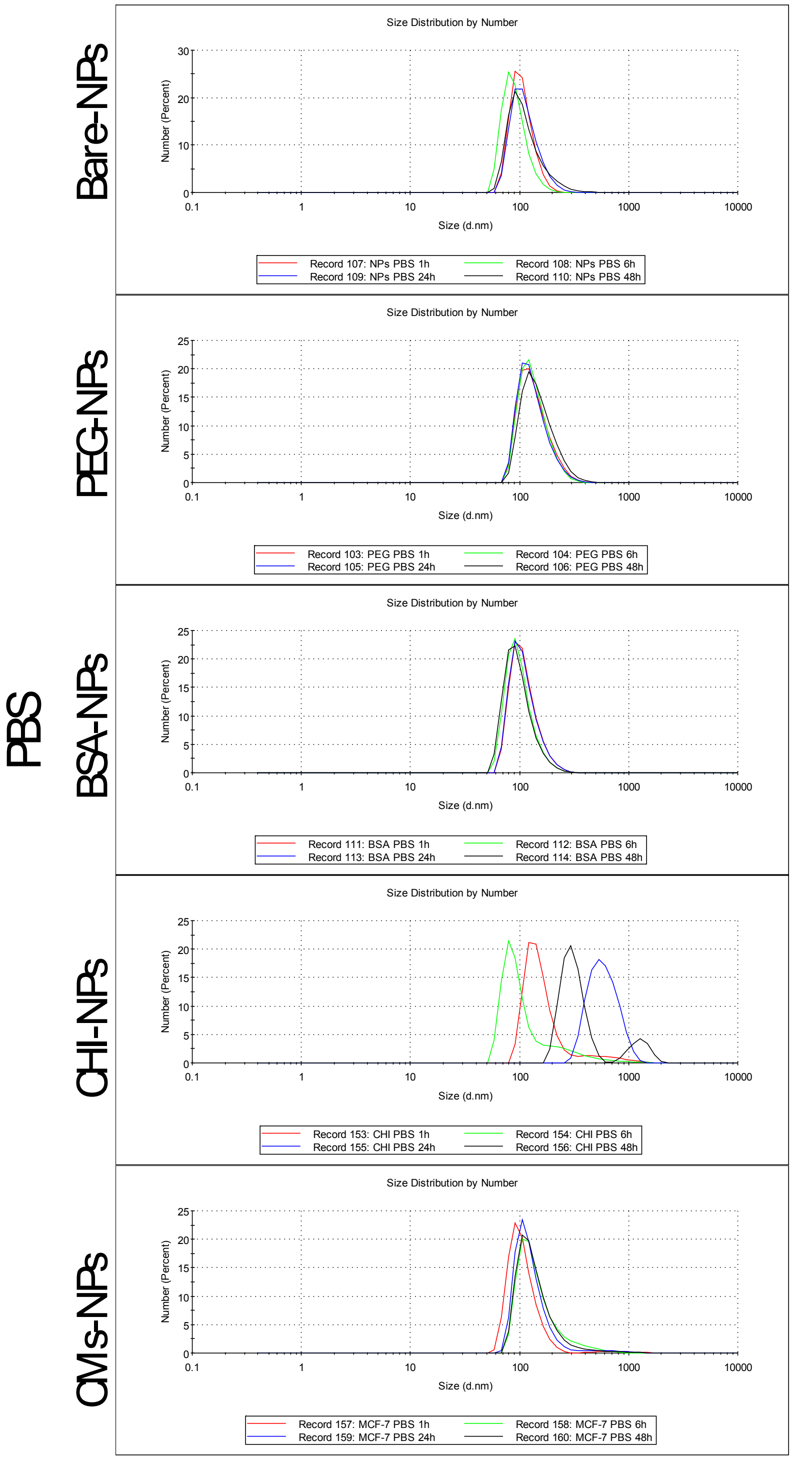

Figure S6. Size distribution by number of the prepared nanosystems in PBS at different time points.

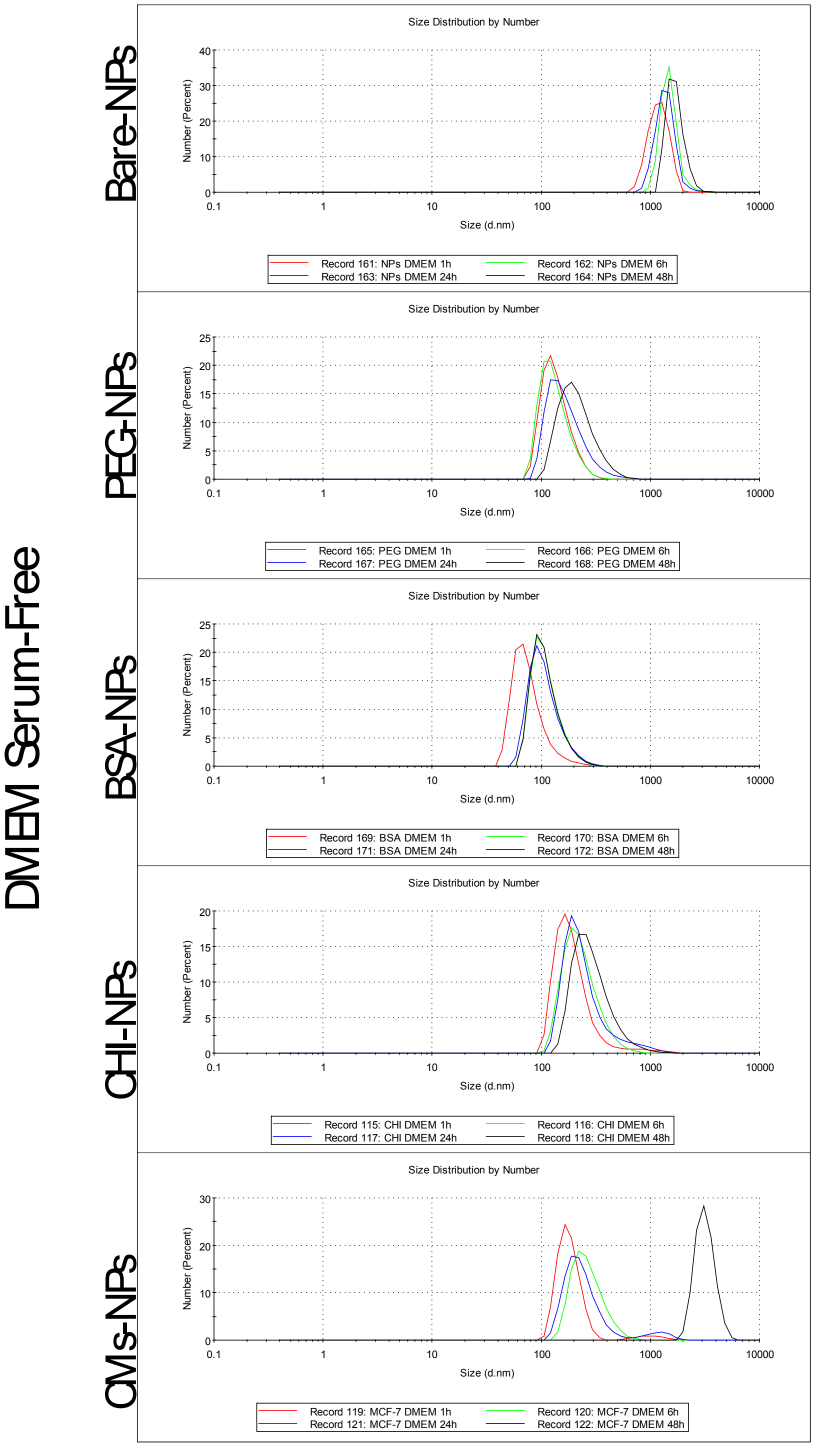

Figure S7. Size distribution by number of the prepared nanosystems in DMEM at different time points.

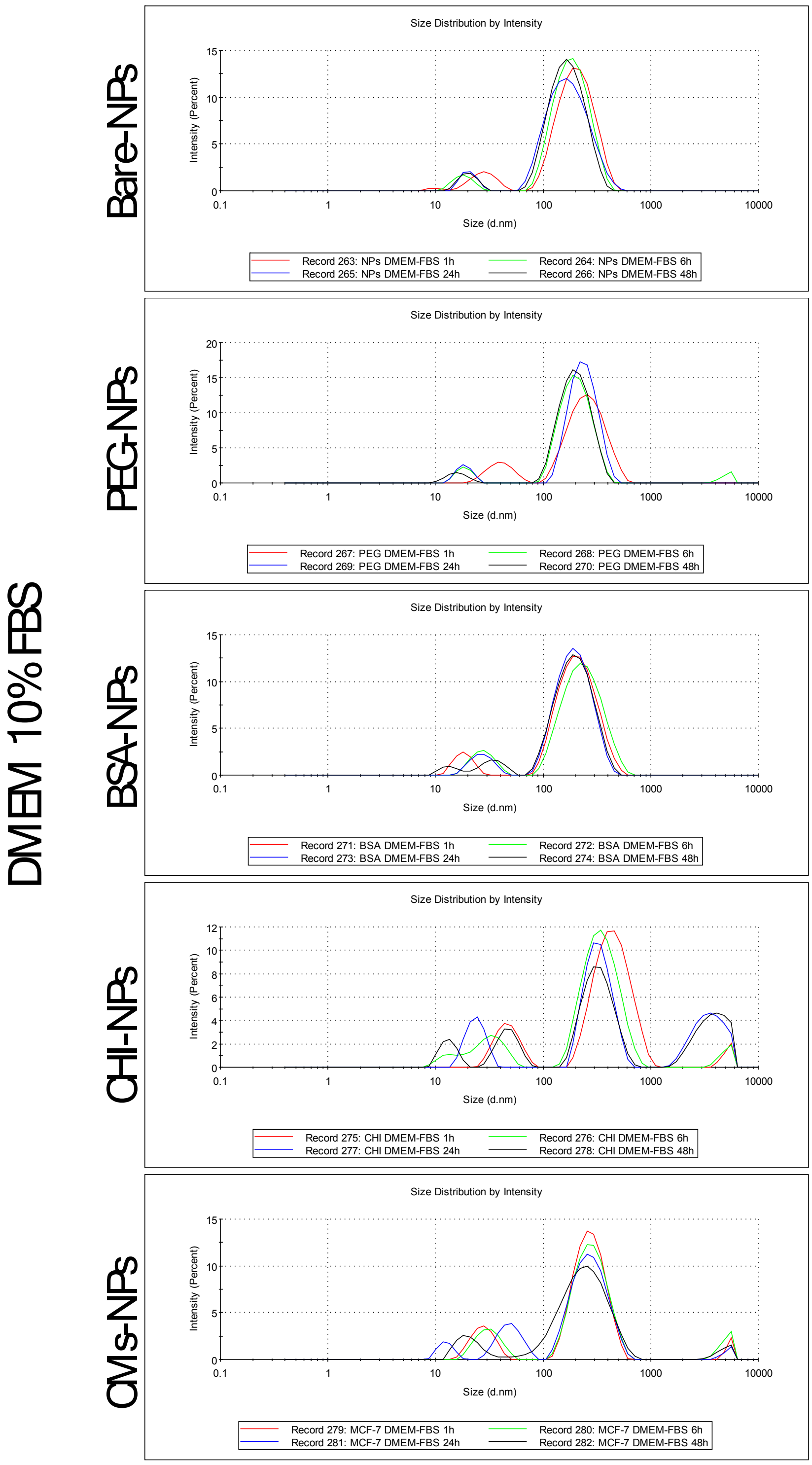

Figure S8. Size distribution by number of the prepared nanosystems in DMEM with 10% FBS at different time points.

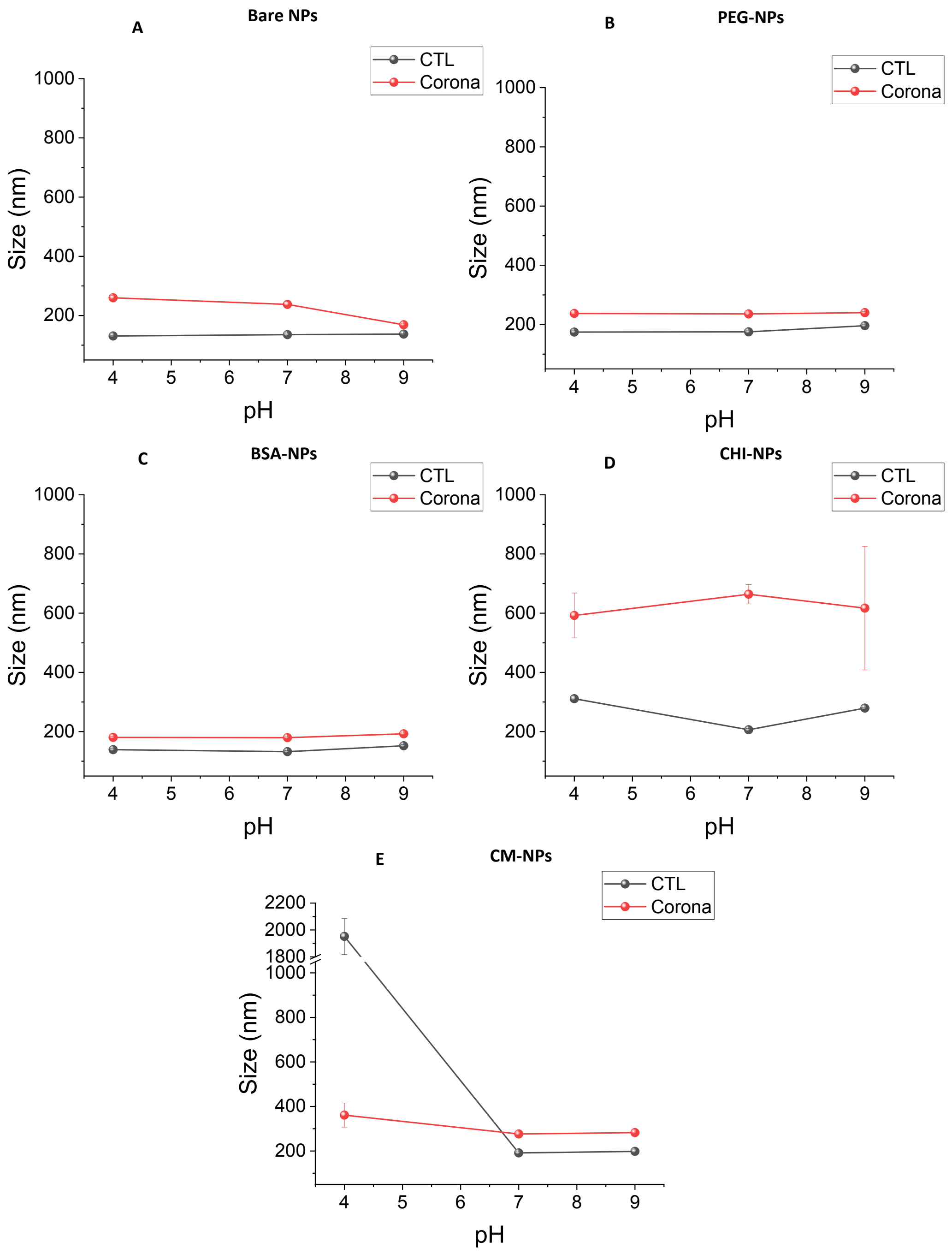

Figure S9. (A-E) Size of the prepared NPs before and after their incubation with complete DMEM and NPs-complexes isolation measured at pH 4, 7 and 9 (n = 3; mean ± SD).

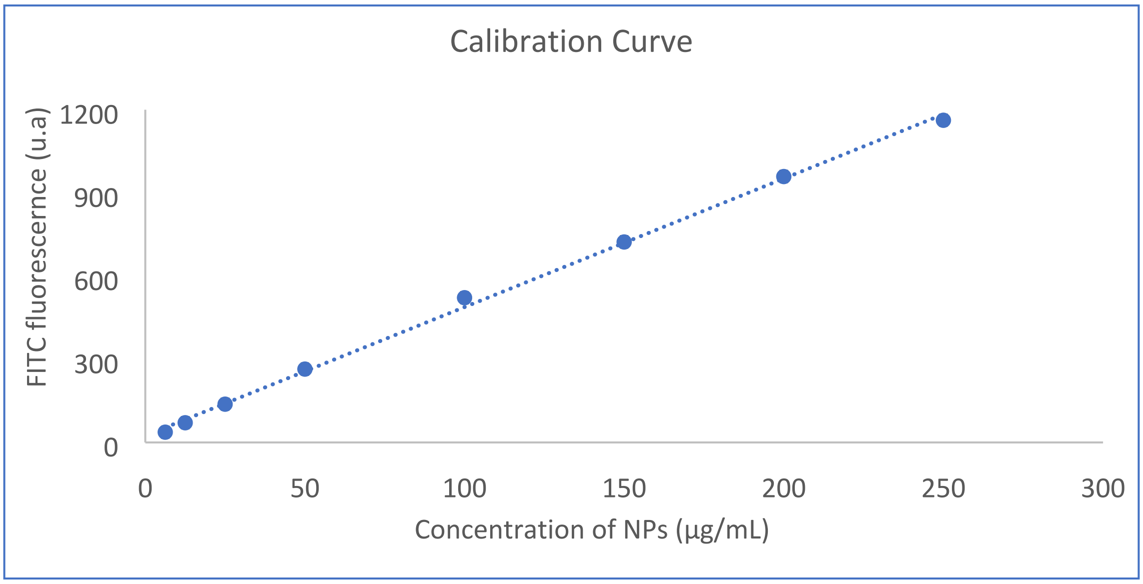

Figure S10. Calibration curves of NPs based on FITC fluorescence.
